# Supplementary material for: Exploring barriers to integrated care for children under 5 living in temporary accommodation: a qualitative study of professionals’ experiences during the COVID-19 pandemic in England
Source: BMJ Open. 2026 Jan 28;16(1):e106318. doi: 10.1136/bmjopen-2025-106318 (PMC12853516; doi:10.1136/bmjopen-2025-106318)
Supplement: Supplementary data [file bmjopen-16-1-s001.pdf]

CHAMPIONS Interview schedule, Professionals v0-6

Opening

- Personal introduction to researcher
- Interview topics to be covered and focus of interview – ie what is different about living in COVID times compared to living in TA before this
- Interview length, format and breaks
- Confidentiality and consent
- Any questions

Scene-setting

Please tell me a little bit about your role:

- What services do you provide to families experiencing homelessness? Is this service specifically for families or also for other groups?
- Does your area have any services specifically for children?
- How do you work with them under normal circumstances?
- How much experience do you have in working with families living in TA? How do you find out about the specific set of problems these children might face?
- What training do staff have received that is relevant to the group they work with. [If they identify anything] Is this provided in house or externally? Did you source it yourself or is it offered by your employer?
- Are you or other members of your team trained or skilled specifically to work with a) children, b) children in TA?
- Are you familiar with your families' accommodation? Do you have any comments about the quality and safety of the environment?

Focus on specific topics

[Follow up questions will be asked to further explore the key topics of interest where they are not covered already by the discussion of photos]

1. Contact and links with other services

Is families' or the child's homelessness status relevant to the service you provide? If so, what do you do differently with them that you don't do with other families, children or client groups?

Can you tell me a bit about how children in your area come to light to the different services and which services? Which organisation or person is responsible for looking after families / children living in TA? Who keeps track of families? What happens if families move?

Are children in TA referred to you: eg is it flagged on an IT system; are you informed, or do you need to ask?

What are the barriers to finding out about their homelessness status and providing appropriate services? (knowledge, skills, access to data, communication internally or with other agencies)

Are there other barriers to providing a service to this group of children and their families? Do you know of any barriers that families experience in accessing your service?

Is your service part of a pathway for families/children to access your service? Who refers them? Do you refer them on to other agencies, and which ones? Do you work in tandem, and how effective is

that? Who follows up to ensure that families continue to access services?

How do these various services link together?

2. COVID Knowledge:

How does your organisation find out about COVID restrictions, and keep up to date with changes? How useful were the sources of information and how well did they apply to your service and service users? Who makes decisions about how to define and when to use safe working practices? How are staff informed about this?

Are you or your team responsible for cascading information about COVID restrictions to families, and how do you go about that? How well has that worked? What worked/what didn't?

3. How has COVID changed the way you work?

We began by asking how you normally go about providing your service.

To what extent do you know, based on your experience, how different are the challenges under covid? How has this changed in terms of:

- Access to families or children to provide your service and the length of time it takes
- Access and referrals to other services such as health visitors, medical care, development checks, dental care, ongoing care for physical/mental health issues
- The way you assess their needs.
- Has the assessment method changed?
    - How have families/children's needs changed since covid? How has your service responded to that?
- Are any of these changes likely to become 'business as usual' for your service delivery?
- Has there been a difference in service delivery between full lockdown and living with COVID restrictions?
- Have you been able to access any additional funding or resources (e.g. staff) to support the service change needed to deal with the pandemic? If so how did that come about, was it a request or it came from (local) government? Do you think it will be sustained beyond 2021?

Do you have any recommendations for what could be done to address these issues?

4. What impact is COVID having on families living in TA? → with examples if possible

From your knowledge of the families, what do you think their biggest concerns have been?

Have these issues affected their ability to engage with [your service]?

What do you think has been the greatest impact on children under 5 living in TA through the COVID pandemic and how has it affected their [health/wellbeing and development/education]? What have your main worries / concerns/observations been?

- Child development (health and social aspects)
- Housing quality/issues and access to (safe) outdoor space/play areas
- Nutrition: cooking and eating
- Education
- Wellbeing/Resilience in general

Pierce PAS, et al. BMJ Open 2026; 16:e106318. doi: 10.1136/bmjopen-2025-106318

- Factors that complicate the situation or make it more difficult, such as disability

Are you aware of the local TA boards within their own LA or borough which discuss how to set up and manage services for homeless families? Is there one locally? Is your service involved with it?

Health visitors:

Thinking about the targets set in the Healthy Child Programme, do you think the pandemic has affected the child's:

- mental health
- physical development
- emotional wellbeing
- diet and access to healthy food

and how? What are the priority areas for recovery?

What support do families need to ensure this happens, and which organisations do you need to work with to provide it?

Health professionals:

Charities:

How much of your service provision relies on fundraising, and has COVID affected this?

Has there been any discussion of the financial and wider sustainability of services? Have any decisions been made about changing the way the service is provided?

Housing teams:

How have you found out about the specific needs of people who use your service?

Have your families needed any additional support from your service, such as

- Signposting or support to access welfare services
- Food banks
- Mental health support

What happens to the child or family after they move on from your service?

What happens if the TA accommodation allocated to a family with children is unsuitable for their needs, for example is overcrowded, has dampness/mould, is in need of repairs, has access issues for prams/buggies, is shared with other unsuitable residents: how would you become aware of these problems (e.g. families complain, or regular property inspections) and what are the options available to you for re-housing them and/or making repairs if appropriate?

Education:

Do you know which pupils are living in TA? Have you identified any specific needs for this group?

Are there any services or approaches that you take to support them?

(access to outdoors and spaces to play)

How has COVID 19 affected the education and school-readiness of this specific group? Does the

school provide additional support (one to one sessions, food parcels, uniform bank, access to IT, printed work pack etc)

Have they been able to access online learning – and what are the barriers to that?

What support do children need during pandemic recovery and afterwards?

Potential solutions

We have discussed your service runs, and how it supports families through the system, and how that has changed during COVID. I want to ask your views on how we can provide a better service for the future, and we set up a system that supports these children.

1. Earlier in our conversation you identified some major issues [such as...] for professionals in your field who are supporting families. What action do you think would address these concerns?
2. We have seen many new innovations and strategies during COVID such as the vaccination bus, the vaccination camps etc. How can we reach families who sometimes find it hard to access services ie how do we bring them services they need closer to them?
3. We are hearing stories about children being 'left behind' following the COVID restrictions, for example low weight, delayed language development and immunisations, and early signs of mental health problems, and there's a view we will need to help children catch up or address problems arising from COVID restrictions. What do you think we should be doing for these children to help them catch up and overcome some of the problems they have gone through?
4. At a national level:
    a. what do you think we should be advocating for to support children - what should our 'ask' be from health, housing, education, local government and central government?
    b. What are the top three actions, or services/facilities that need to be put in place, that you would like to see?
    c. What are your views on setting up an integrated care package around each child?
    d. What are your views on setting up a national notification system?
    e. Who are the key stakeholders in this process?

Closing

Is there anything else you would like to add that we haven't discussed so far?

If you'd like to add any further thoughts or comments, then please do so by contacting me at the email address or postal address on the information sheet.

At the end of the project we will write a summary of the results and send you a copy if you wish.

Thank you for taking part in this research.
