# Supplementary material for: Exploring barriers to integrated care for children under 5 living in temporary accommodation: a qualitative study of professionals’ experiences during the COVID-19 pandemic in England
Source: BMJ Open. 2026 Jan 28;16(1):e106318. doi: 10.1136/bmjopen-2025-106318 (PMC12853516; doi:10.1136/bmjopen-2025-106318)
Supplement: Supplementary data [file bmjopen-16-1-s002.pdf]

COREQ (COnsolidated criteria for REporting Qualitative research) Checklist

A checklist of items that should be included in reports of qualitative research. You must report the page number in your manuscript where you consider each of the items listed in this checklist. If you have not included this information, either revise your manuscript accordingly before submitting or note N/A.

Topic	Item No.	Guide Questions/Description
Interviewer/facilitator	1	Which author/s conducted the interview or focus group? **Methods, Materials section. Acknowledgments section.**
Credentials	2	What were the researcher's credentials? **Methods, Materials section.**
Occupation	3	What was their occupation at the time of the study? **Methods, Materials section.**
Gender	4	Was the researcher male or female? **N/A**
Experience and training	5	What experience or training did the researcher have? **Methods, Materials section.**
Relationship established	6	Was a relationship established prior to study commencement? **Methods, Materials section.**
Participant knowledge of the interviewer	7	What did the participants know about the researcher? e.g. personal goals, reasons for doing the research. **Methods, Materials section.**
Interviewer characteristics	8	What characteristics were reported about the interviewer/facilitator? e.g. Bias, assumptions, reasons and interests in the research topic. **Methods, Materials section.**
Methodological orientation and Theory	9	What methodological orientation was stated to underpin the study? e.g.grounded theory, discourse analysis, ethnography, phenomenology, content analysis. **Analysis section.**
Sampling	10	How were participants selected? e.g. purposive, convenience, consecutive, snowball **Methods, Participants section.**
Method of approach	11	How were participants approached? e.g. face-to-face, telephone, mail, Email. **Methods, Participants section.**
Sample size	12	How many participants were in the study? **Results section.**
Non-participation	13	How many people refused to participate or dropped out? Reasons? **N/A**
Setting of data collection	14	Where was the data collected? e.g. home, clinic, workplace **Methods, Materials section.**
Presence of non-participants	15	Was anyone else present besides the participants and researchers? **Methods, Materials section.**
Description of sample	16	What are the important characteristics of the sample? e.g. demographic data, date demographic data. **Results section.**
Interview guide	17	Were questions, prompts, guides provided by the authors? Was it pilot-tested? **Methods, Materials section.**
Repeat interviews	18	Were repeat interviews carried out? If yes, how many? **Methods, Materials section.**
Audio/visual recording	19	Did the research use audio or visual recording to collect the data? **Methods, Materials section.**
Field notes	20	Were field notes made during and/or after the interview or focus group? **Methods, Materials section.**
Duration	21	What was the duration of the interviews or focus group? **Methods, Materials section.**
Data saturation	22	Was data saturation discussed? **Methods, Participants section.**
Transcripts returned	23	Were transcripts returned to participants for comment and/or correction? **N/A**

Topic	Item No.	Guide Questions/Description
Number of data coders	24	How many data coders coded the data? **Analysis section.**
Description of the coding tree	25	Did authors provide a description of the coding tree? **Analysis and Results section.**
Derivation of themes	26	Were themes identified in advance or derived from the data? **Analysis section.**
Software	27	What software, if applicable, was used to manage the data? **Analysis section.**
Participant checking	28	Did participants provide feedback on the findings? **N/A**
Quotations presented	29	Were participant quotations presented to illustrate the themes/findings? Was each quotation identified? e.g. participant number **Results section.**
Data and findings consistent	30	Was there consistency between the data presented and the findings? **Results and Discussion section.**
Clarity of major themes	31	Were major themes clearly presented in the findings? **Results section.**
Clarity of minor themes	32	Is there a description of diverse cases or discussion of minor themes? **Results section.**

Developed from: Tong A, Sainsbury P, Craig J. Consolidated criteria for reporting qualitative research (COREQ): a 32-item checklist for interviews and focus groups. *International Journal for Quality in Health Care*. 2007. Volume 19, Number 6: pp. 349 – 357

Once you have completed this checklist, please save a copy and upload it as part of your submission. DO NOT include this checklist as part of the main manuscript document. It must be uploaded as a separate file.
