# Supplementary material for: Exploring barriers to integrated care for children under 5 living in temporary accommodation: a qualitative study of professionals’ experiences during the COVID-19 pandemic in England
Source: BMJ Open. 2026 Jan 28;16(1):e106318. doi: 10.1136/bmjopen-2025-106318 (PMC12853516; doi:10.1136/bmjopen-2025-106318)
Supplement: Author's manuscript [file bmjopen-2025-106318.draft_revisions.pdf]

BMJ Open is committed to open peer review. As part of this commitment we make the peer review history of every article we publish publicly available.

When an article is published we post the peer reviewers' comments and the authors' responses online. We also post the versions of the paper that were used during peer review. These are the versions that the peer review comments apply to.

The versions of the paper that follow are the versions that were submitted during the peer review process. They are not the versions of record or the final published versions. They should not be cited or distributed as the published version of this manuscript.

BMJ Open is an open access journal and the full, final, typeset and author-corrected version of record of the manuscript is available on our site with no access controls, subscription charges or pay-per-view fees (http://bmjopen.bmj.com).

If you have any questions on BMJ Open's open peer review process please email info.bmjopen@bmj.com

BMJ Open

Barriers to integrated care faced by professionals supporting children under 5 in temporary accommodation, lessons from the COVID-19 pandemic

Journal:	*BMJ Open*
Manuscript ID	bmjopen-2025-106318
Article Type:	Original research
Date Submitted by the Author:	10-Jun-2025
Complete List of Authors:	Pierce, Poppy; University College London Faculty of Medical Sciences, Medical School<br>Svirydzenka, Nadzeya; De Montfort University Faculty of Health and Life Sciences, Applied Social Sciences<br>Adil, Haleema; University College London Faculty of Medical Sciences, Medical School<br>Allaham, Shereen ; UCL GOS ICH, Population, Policy and Practice Research and Teaching Department<br>Ankers, Matthew; Flinders University College of Nursing and Health Sciences,<br>Parry, Yvonne; Flinders University, School of Nursing & Midwifery<br>Heys, Michelle; UCL GOS ICH, Population, Policy and Practice Research and Teaching Department; East London NHS Foundation Trust, Specialist Children's and Young People's Services<br>Ucci, Marcella; University College London, Bartlett School of Graduate Studies<br>Lakhanpaul, Monica; UCL GOS ICH, Population, Policy and Practice Research and Teaching Department; Nottingham University Hospitals NHS Trust, Community Paediatrics
Keywords:	Delivery of Health Care, Integrated, Family, COVID-19, Health Services, Community child health < PAEDIATRICS, Vulnerable Populations

SCHOLARONE™
Manuscripts

I, the Submitting Author has the right to grant and does grant on behalf of all authors of the Work (as defined in the below author licence), an exclusive licence and/or a non-exclusive licence for contributions from authors who are: i) UK Crown employees; ii) where BMJ has agreed a CC-BY licence shall apply, and/or iii) in accordance with the terms applicable for US Federal Government officers or employees acting as part of their official duties; on a worldwide, perpetual, irrevocable, royalty-free basis to BMJ Publishing Group Ltd ("BMJ") its licensees and where the relevant Journal is co-owned by BMJ to the co-owners of the Journal, to publish the Work in this journal and any other BMJ products and to exploit all rights, as set out in our licence.

The Submitting Author accepts and understands that any supply made under these terms is made by BMJ to the Submitting Author unless you are acting as an employee on behalf of your employer or a postgraduate student of an affiliated institution which is paying any applicable article publishing charge ("APC") for Open Access articles. Where the Submitting Author wishes to make the Work available on an Open Access basis (and intends to pay the relevant APC), the terms of reuse of such Open Access shall be governed by a Creative Commons licence – details of these licences and which Creative Commons licence will apply to this Work are set out in our licence referred to above.

Other than as permitted in any relevant BMJ Author's Self Archiving Policies, I confirm this Work has not been accepted for publication elsewhere, is not being considered for publication elsewhere and does not duplicate material already published. I confirm all authors consent to publication of this Work and authorise the granting of this licence.

For peer review only

Title: Barriers to integrated care faced by professionals supporting children under 5 in temporary accommodation, lessons from the COVID-19 pandemic

Corresponding author

Monica Lakhanpaul, m.lakhanpaul@ucl.ac.uk, +447775506418

Authors' affiliations
Poppy Pierce* - Medical School, Faculty of Medical Sciences, University College London, London, United Kingdom poppy.pierce.19@ucl.ac.uk
Nadzeya Svirydzenka* - School of Applied Social Sciences, Faculty of Health and Life Sciences, De Montfort University, Leicester, United Kingdom nadzeya.svirydzenka@dmu.ac.uk
Haleema Adil - Medical School, Faculty of Medical Sciences, University College London, London, United Kingdom haleema.adil.19@ucl.ac.uk
Shereen Allaham - Population, Policy and Practice Research and Teaching Department, University College London Great Ormond Street Institute of Child Health, London, United Kingdom s.laham@ucl.ac.uk
Matthew Ankers - College of Nursing and Health Sciences, Flinders University, South Australia matthew.ankers@flinders.edu.au
Yvonne Parry - Caring Futures Institute, Flinders University, South Australia yvonne.parry@flinders.edu.au
Michelle Heys - Population, Policy and Practice Research and Teaching Department, University College London Great Ormond Street Institute of Child Health, London, United Kingdom m.heys@ucl.ac.uk

- Specialist Children's and Young People's Services, Newham, East London NHS Foundation Trust

Marcella Ucci - Institute for Environmental Design and Engineering, Bartlett Faculty of the Built Environment, University College London, London, United Kingdom m.ucci@ucl.ac.uk
Monica Lakhanpaul** - Population, Policy and Practice Research and Teaching Department, University College London Great Ormond Street Institute of Child Health, London, United Kingdom m.lakhanpaul@ucl.ac.uk

- Community Paediatrics, Nottingham University Hospitals NHS Trust

*Poppy Pierce and Nadzeya Svirydzenka are joint first authors
**Monica Lakhanpaul is the senior author

Author Contributions
PP - Data Curation, Analysis, Writing (Original Draft Preparation)
NS - Conceptualisation, Funding Acquisition, Methodology, Supervision, Analysis, Writing (Review & Editing)
HA - Analysis, Writing (Original Draft Preparation)
SA - Supervision, Data interpretation, Writing (Review & Editing)
MA - Data interpretation, Writing (Review & Editing)
YP - Data interpretation, Writing (Review & Editing)
MH - Conceptualisation, Funding Acquisition, Writing (Review & Editing)
MU - Conceptualisation, Funding Acquisition, Writing (Review & Editing)
ML - Conceptualisation, Funding Acquisition, Project Administration, Methodology, Supervision, Analysis, Writing (Review & Editing)

For peer review only

Funding: ESRC as part of UK Research & Innovation's rapid response to COVID-19. No.ES/V016253/1

Acknowledgments: The authors wish to thank all of the professionals who took the time out of their busy schedules to take part in these interviews, especially given the challenging circumstances in which data was collected.

We would like to acknowledge the hard work of researchers that supported the development of the project ethics and data collection - Dr Margarita Garfias Royo and Dr Kriss Fearon respectively.

We would also like to thank all our partner organisations who supported CHAMPIONS and all the participants who kindly gave their time to support the study.

We would also like to thank Dr Diana Margot Rosenthal for her contribution to the conceptualisation of the project, writing of the CHAMPIONS project proposal, and sharing of insights from her PhD research on health and healthcare service access for children under 5 living in temporary accommodation due to experiencing homelessness.

Finally, the authors would like to thank UKRI/ESRC for funding this important work.

Conflicts of Interest: The authors declare no conflict of interest.

Data availability statement: The data that support the findings of this study are available from the corresponding author upon reasonable request.

Key words: Integrated systems; family homelessness; child homelessness; temporary accommodation; COVID-19; policy and practice

Abstract

Background: Homelessness among families in the UK has reached critical levels, with children under 5 in temporary accommodation (TA) facing heightened risks to their health and development, due to unstable living conditions and limited access to services. The COVID-19 pandemic exposed the vulnerabilities in care systems, offering a unique insight into the barriers to cross-sector collaboration in delivering sustainable, integrated support for this vulnerable group.

Methods: Between September 2021 and March 2022, 45 semi-structured interviews were conducted with professionals across health, housing, education, and non-profit sectors in England.

Results: This study highlights the complex, multi-level barriers that professionals face when delivering integrated care to children under 5 in TA. Key barriers emerged across four interconnected levels: individual, team, work environment, and institutional context. From siloed working practices and limited training to staffing shortages and restrictive data-sharing policies, these challenges collectively hinder service continuity and collaboration.

Conclusion: Although this project was conducted during the COVID-19 pandemic, the challenges identified reflect deeper, long-standing issues in service delivery. As services continue to recover and prepare for future crises, these insights remain highly relevant and can inform more resilient, integrated recovery plans to support children in TA beyond the pandemic context. Addressing these barriers, through improved collaboration, training, and data-sharing, is key to strengthening care for this vulnerable population.

Article summary
Strengths and limitations of this study

- The study included diverse professionals from health, housing, and social care across England, enhancing relevance and transferability.

- Semi-structured interviews were conducted with a detailed topic guide, allowing for consistency and rich, nuanced data collection.
- Limited staff availability due to the COVID-19 pandemic led to opportunistic rather than representative sampling.
- The study could not account for local variation in services or enable comparative regional analysis.

Word count: 6017

Introduction

Family homelessness is an escalating issue within high-income countries, including the United States and across Europe (1). In the UK, this trend is particularly alarming, with over 164,000 children living in temporary accommodation (TA) with their families in 2024; an increase of 15% since 2023 (2). The rising numbers reflect the deepening housing crisis, exacerbated by economic pressures, a lack of liveable housing stock, and inadequate access to affordable housing (3). The 2025 Shelter Scotland report provides stark evidence of how living in TA harms children's health, development, and education, reinforcing the urgent need for cross-sector action to prevent long-term consequences (4). The COVID-19 pandemic saw a sharp increase in the number of families living in TA across England, impacting an estimated 253,000 individuals (5,6). Children under 5 were particularly susceptible to poor health outcomes, due to limited healthcare access; unstable and substandard living conditions; and the disruption to early development (7-9). The COVID-19 pandemic inadvertently served as a stress test for public services, showcasing effective practices but also exposing pressing issues within these systems (10). Addressing these enduring challenges is urgent, as this is a critical period of rapid growth for children under 5, where timely intervention can prevent long-term developmental challenges and offer a unique opportunity to positively shape a child's futures (7,11,12).

The COVID-19 pandemic significantly disrupted care systems worldwide but also compelled health and social care partners to collaborate more closely and rapidly than they had previously (13,14). Professionals reported improved cross-organisational working as priorities aligned to address the crisis (15). Integrated community health responses and initiatives such as 'Everyone In' helped maintain care access and positioned homelessness as a public health priority (16,17). However, children were largely overlooked in COVID-19 policy and planning, with low-income families with children under 5 being disproportionately disadvantaged (18,19). The pandemic highlighted numerous vulnerabilities in the systems that support children under 5 in TA, such as staff shortages, limited training, and communication breakdown between teams (12). Following the pandemic, many of these issues remain unresolved, with additional challenges like the cost of living, austerity measures, and the housing crisis continuing to hinder recovery (20-23). The result is overlapping crises which are also likely to be exacerbating each other. As we anticipate future crises, it is imperative to address healthcare provision for children under 5 in TA and how to sustain such provision in a system under stress. The majority of children under 5 in TA face complex and multifaceted challenges, where solutions do not necessarily lie within the remit of a singular organisation (24,25). Effective cross-sectoral action is needed to meet current demands, and the work to recover, prepare, and innovate must be framed through a health equity lens (25-28).

Although research on children under 5 in TA during the pandemic is scarce, one study conducted in Newham, an exceptionally deprived and diverse inner-city London borough, offers valuable local insight into professionals' perspectives on the barriers families in TA faced in accessing healthcare during this time (12). While the previous study focused on

professionals' perspectives on families' challenges within a single borough, this study shifts the lens to professionals' reflections on their own roles and the barriers to cross-sector collaboration at a national level. In doing so, it builds on and extends existing knowledge, addressing a critical gap in understanding how to improve cross-sector working to better support children under 5 in TA. This research aims to qualitatively explore the factors that hinder professionals in delivering integrated care for children under 5 in TA and understand their experiences of collaboration during the pandemic to inform recommendations. The recommendations follow two key objectives: (i) to explore cross-sector learnings that are applicable to improving integrated care; (ii) tailor recommendations to the needs of families and children under 5 experiencing homelessness in the UK today.

Methods

This study sits within the CHAMPIONS programme of research which focuses on the impact of living in poverty on children under 5 living in TA (29). During the COVID-19 pandemic, a mixed method multi-phased study was conducted with the specific aim of understanding the experiences of children in TA during that time. This paper represents only the work package dedicated to the qualitative interviews with professionals engaged with and supporting the cross system around the child and their families.

Participants

Purposive and snowball sampling was employed (30,31) to recruit a representation of key professionals across England. Those not eligible to take part in the study included people who did not work with families and/or children in TA settings. No participants were excluded based on protected characteristics or any demographic factors. Participants were recruited through a variety of channels, including:

- Housing, welfare, refugee, and domestic violence support organisations, as well as housing providers such as local authorities and housing associations.
- Direct referrals from professionals, including health visitors and housing officers.
- Social media platforms and WhatsApp groups managed by the project team and partner support organisations.
- Leaflets and posters distributed through food banks and family support charities.
- Snowballing through word of mouth.

Recruitment was undertaken between September 2021 and March 2022. Participants were identified professionals within key areas who were invited to take part in the study via an email, leaflet, or word of mouth. All potential participants were asked to email the researchers to indicate their interest in taking part. Interested participants received an information sheet and were encouraged to respond with any questions, or to arrange an interview. Informed consent was obtained prior to participation. Research fellow on the project continued with recruitment and data collection until thematic saturation was reached, which occurred after 45 interviews (32). Despite the challenges of conducting research during the COVID-19 pandemic, the study achieved a strong sample size for qualitative research as data saturation was reached (33). Effective recruitment was facilitated by strong participant engagement strategies, established trust, and the research team's experience and credibility in the field.

Materials

For peer review only

The interview schedule was developed by looking into key domains of supporting families with children under 5 in TA. To gather detailed insights into the perceived barriers to delivering integrated care during the pandemic, the questions addressed topics such as training related to supporting children under 5 in TA; workload and capacity; workplace dynamics and collaboration between sectors, particularly between health, non-profit, and housing services; impact of COVID-19 pandemic on families living in TA; the impact of policies and funding on service delivery; personal approaches to providing care; and recommendations for improving integrated care for children under 5 in TA. Topic guide can be found in Supplementary File 1. A demographic questionnaire was also given to participants to complete prior to the interviews.

The research fellow arranged individual semi-structured interviews with participants, conducted remotely via Zoom (34). All interviews were carried out in English, recorded using Zoom's digital platform. Transcription was carried out by the research fellow or professional transcriber and quality cross checked with the audio file.

COREQ found in Supplementary File 2.

Procedure

Ethics approval was obtained by the UCL Research Ethics Committee (ID number: 9277.004).

Patient and Public Involvement: Patients or the public were not involved in the design, or conduct, or reporting, or dissemination plans of this research.

Analysis

All interview transcripts were anonymised and given a four-digit number code. Researchers PP and HA carried out data analysis using an inductive approach to generate codes through data familiarisation. They were supported throughout by senior team members, including a paediatrician with in-depth knowledge of health and social care systems and academic researchers with expertise in qualitative methods and the relevant subject matter, which strengthened the rigour and interpretation of the findings. Two interviews were co-coded independently and codes were then compared to ensure replicability and reliability. In cases where conflict in codes arose, consensus discussions took place and codes were revised. In the final stage, the codes were organised into four broad themes i.e., individual, team, work, and institutional barriers. Codes were subsequently categorised into sub-themes that emerged in analysis. This followed Braun and Clarke's 6-step framework for qualitative analysis (35). NVivo software (v.12) was used to assist in the organisation and analysis of the data (36).

Results

In total, 45 interviews were conducted. The average time for an interview was 1 hour. Figure 1 shows participant characteristics. Professionals included 9 Health Visitors, 16 Health Professionals, 10 professionals from Non-Profit organisations, 8 Housing Sector professionals and 2 professionals working in education. The participants' ages ranged from 26 to 68 years, with a mean age of 44. The majority were female (39/45), and they had experience working with families living in TA for periods ranging from 1 to over 40 years, with an average of 8 years.

For peer review only

Figure 1: Table showing participant characteristics including role, age, gender, ethnicity, and length of time in role.

Roles of participants interviewed

The professionals interviewed in this study represented a diverse range of roles across healthcare, housing, education, and the charity sector, all working to support children under 5 living in TA in England. Healthcare professionals included GPs, health visitors, midwives, nurses, dentists, a paediatrician, and a speech and language therapist, many specialising in homelessness, safeguarding, refugee support, and perinatal care. Housing professionals, including caseworkers, support workers, solicitors, and housing directors, highlighted that they provided advice on housing rights, challenged poor living conditions, and liaised with local authorities. Educators, such as an early years teacher and a nursery director, supported children's development in challenging circumstances. Non-profit professionals including charity directors, coordinators, peer support leads, play workers, policy advisors, and volunteers, highlighted their focus on direct service delivery, emotional and educational support, advocacy, and strategic planning to address the complex needs of families in TA.

Barriers

The results of this study identified a range of barriers affecting professionals' ability to deliver integrated care for children under 5 living in TA. These themes emerged across four interconnected levels: individual, team, work environment, and institutional context (Figure 2).

Figure 2. Summary of the themes and sub-themes representing barriers to integrated care.

1. Individual factors

1.1 Lack of training and knowledge

The lack of accessible and relevant training was identified by participants as a significant barrier. Professionals highlighted a siloed approach within professional development systems, where they reported limited opportunities to learn how to support families in TA, as well as how to collaborate with other professionals and understand their roles within the wider system:

> *"In terms of ensuring there is adequate care or adequate access for those who are homeless, no training whatsoever." (1132)*

> "*We have a range of a range of expertise within the team, but nobody who's specifically qualified or trained to do work in that specific area…which can be stressful." (3166)*

> "*No, no, not specifically [training], but they [homeless families] do, I would say, cover quite a large proportion of our caseload…One of the things that we find difficult [without training] is negotiating how it works within local authorities to get the support and the housing. And what is allowed and what isn't, and yeah it's a bit sticky, it's a bit of a minefield…these things change quite frequently as well, like they do for everything." (3058)*

For peer review only - http://bmjopen.bmj.com/site/about/guidelines.xhtml

"From what I found when I've looked for training courses for my staff, there is not a lot widely available... especially not in the field of working with children who are facing multiple adversity." (2776)

Instead, teams often relied on informal, experience-based knowledge transfer:

"I think we've probably arrived at that [knowledge about supporting homeless children] by a mix of different things and a bit of learning on the job. But yeah, I'm not aware of anything specific that we've done... Young children especially tend to get a bit forgotten in the whole homelessness issue.. there's a gap and obviously we've got our kind of health visitors and things like that, again, I wouldn't be confident about their level of understanding really when it comes to homelessness because it is a really complicated picture." (4048)

"I wouldn't say I have any specific training at all, it's more just experience and a general interest in looking after people who are I guess more socio economically deprived." (1388)

Moreover, this reported absence of training led to professionals stating they felt unprepared, possibly creating bottlenecks in service delivery particularly during the pandemic. As one participant reflected:

"Our team, we did feel a bit out of our depth at one point." (7114)

1.2 Individual constraints to upskill

Another significant reported barrier was the lack of time and capacity within roles to reflect or upskill which prevented professionals from moving beyond reactive, day-to-day duties to adopt a more strategic approach:

"I think it [training] would be useful, it would be useful. I suppose in some sort of format, it's difficult to imagine how that would look for us as GPs, because I just don't know how that would look in terms of time, I suppose." (1388)

"And then we're at the stage now we need a little bit more calm and capacity, a bit more head space. Twenty percent of our staff are off sick at the moment, so you try and put a training program together and it doesn't make sense. They're just on survival mode. So wait for the pandemic to die down and then the recovery period, probably in the spring summer, it's a little quieter, we will then put together, put our heads together. And look at ways in which we can address this. Either through some professional educational input, which goes to everybody, and we have to think again how to do it. But at the moment there's just no space, head space to do it." (4828)

2. Team factors

2.1. Misaligned goals between teams

A fundamental challenge emerged around the differing priorities between teams, which reportedly hindered collaboration and reduced the effectiveness of integrated support. Professionals reported that while health professionals considered housing a critical determinant of health, housing officers focused solely on their legal duties:

"Because for a housing officer, it's not really on their agenda, whether they've been for a smear test or whether the baby's had immunisations or whether or not that family is practicing safe sleep, all of these prevention things. It's not really their... it's not their priority. Their priority is to look at the legal duty that has been offered to that family... Housing Officers do not see that health is part of what will solve their problems." (1151)

This divergence in priorities was compounded by systemic fragmentation between teams:

"We all got different funding. We're all different agencies and nobody talks to each other. And that's the key problem. And it's like that with the NHS actually isn't it I mean that the left hand never talks to the right everything needs to come under one umbrella really when it comes to children because it's a whole process. You can't just have one thing being done. And the other bit nuts it's like having a jigsaw and not being able to finish it." (6555)

Furthermore, professionals described difficulties in securing buy-in from leadership across teams, reinforcing silos in service provision:

"Sometimes you're asked during professional development or during your one to one's, what kind of professional development do you need, what will help you better to do your job properly. How can you help our residents better? And I would make suggestions and it's almost like well, we've tried that, we're not getting anywhere because the other team managers are not cooperating. So, it's almost like there's a barrier because we have tried…but you can't get the buy in from the other departments because there's a, it's just as bureaucracy and barriers… If people don't buy into a certain practice, and it comes from leadership really." (2226)

2.2. Poor communication between teams

Limited communication was a recurring issue, reported as contributing to inefficiencies and delays in service provision. Many professionals described that they found themselves acting as intermediaries due to fragmented communication channels:

"So then we become sort of mediators between what customer services cannot do and between back-office housing where they don't have that direct contact. Because even the phone lines and the emails, I think due to demand, in my opinion, it's almost like there's a barrier there. They can't access housing advisors, officers, housing officers directly. Unless sometimes by luck, the phone, somebody picks up or somebody decides they're going to email them. Because they might send them one email and six months later they hear back. But they are all irregular or poor communication." (2226)

"the local authority decided to move these children from some of the homeless units to another homeless accommodation, either within the borough or outside the borough. So again, it took a long long time trying to trace these children because they disappear over, we don't know where they moved to." (1988)

Professionals also highlighted that escalation of their concerns via standard, recommended routes, such as commissioner intervention, were ineffective:

For peer review only

"We've even had emails from commissioners saying oh if they do that, if they move somebody without letting you know, if you request for delayed dispersal and they disperse the person [then] this is the person to email. And we continually do that but nothing happens, it still happens, it still happens yeah." (4029)

3. Work environmental factors

3.1. Reduced staffing levels and high staff turnover

Staffing shortages were highlighted as a significant barrier, with professionals describing burnout and high turnover rates as contributing to inefficiencies.

"There's a lot of mandatory training you have to do to work for our trust. So the bank staff, we did hire, our recruitment time takes like, three, four months. It's a really, really long, arduous process. So that's the first part. And the second part where they've now asked, Does anyone want to do Saturdays or evenings? Everyone's burnt out - no." (4999)

Many teams were described as not able to access any additional funding or extra staff to help with the increased workload during the pandemic:

"No. If anything it's less [funding], because you'd have to cover, as times gone on people are shielding or not shielding and all the rest of it. You're covering your colleagues work, staff are just ill, they're burned out, they're stressed. So staffing has been a huge, huge issue, much worse. And lots of people have been deployed as well." (5186)

3.2. Workload and capacity issues

High caseloads and limited capacity were key reported concerns, particularly in urgent care settings where referral processes created bottlenecks:

"These urgent care centres will receive a walk in or a phone call from a patient asking to be seen, a child, and before even assessing the child they just send them straight to the community referral service. Which then creates a huge backlog for these community dental services. And it's just, it's very, very unreasonable when these children could actually be treated at the urgent care centres." (1132)

Participants described the backlog in services, which was exacerbated by the pandemic, as further restricting access to routine and preventative care:

"Very difficult at the moment, because we have such a huge backlog because of the pandemic, not being able to see patients routinely for so long. We're still getting through our backlog, there are still patients who I've seen this week who haven't been seen for two years and they are registered in our books. So our registration is closed at the moment, so we're not taking on any new patients. So we can see them on an emergency basis, but not to take on to give them that preventative care, to give them that routine care. It's just to get them out of pain only." (1132)

4. Institutional context factors

For peer review only

4.1. Lack of funding and senior support

Professionals highlighted how funding structures and reliance on senior support at the institutional level posed barriers to their ability to deliver integrated, preventative care for young children in TA. In particular, professionals highlighted the way service payment models in dentistry actively disincentivised early intervention:

> *"If you have a child under the age of two, you're not really going to be doing any treatment on them. So, the dentist, the dental practice doesn't get paid to have that preventative chat with the patients. So there's no financial incentive of seeing patients under the age of two, so I think that dentists don't see patients under the age of two." (1132)*

Participants felt that preventative care was also hindered by a reliance on strategic leadership buy-in at the institutional level:

> "*Unless the officer, the managers and the, the higher, the chief officers, are aware that we need to do some prevention work to stop these families coming back into the homeless system - what we know is if babies and children are in the homeless system we know that they'll be more likely to be adults that will be within the homeless system. So it's really the open mindedness of those strategic leads to see that if we can implement and step in with prevention.*" (1151)

4.2. Policy constraints and catchment area limitations

Service provision was described as highly inconsistent due to catchment area policies:

> *"So some children you just lose track of them because they don't ever get back…when it's impossible, for whatever reason, in exceptional circumstances we will retain that care just to keep the continuity going for the child. Despite the fact that it might be outside a contract of some sort. These are the constraints that really these poor families in a way are up against, institutional constraints. Not because the clinician doesn't want to see them but because the institution rules are such that they've now moved out, they need to go somewhere else… we've been prohibited by an institutional rule as opposed to what's clinically in the best interest of the child." (4828)*

The data suggested that differences in eligibility criteria between local authorities and charities created frustration, as some individuals fell outside the support systems in place:

> *"There's obviously cases where the local authority, particularly, that obviously they have an eligibility criteria, and some of the people that we support will at times fall outside of that where there can be frustration, I think, as a charity, because we kind of want to help anybody who's experiencing homelessness, and the local authority and some other charities in the city have different criteria around who they work with." (4048)*

4.3. Lack of specialised services

Participants were concerned that the absence of dedicated services for families in TA meant that existing services often failed to meet their needs:

For peer review only

"I don't think one could be optimistic unless you redesign the service for those families. You have to understand their needs and I think health visitors are in a fantastic position to do that. And then you have to be able to define a response to those needs that actually is contracted specifically. It's not going to happen within the existing services, they will fail. We will fail these families, I know that." (4828)

4.4. Data sharing and access issues

Many professionals reported that data protection regulations, such as the UK General Data Protection Regulation (GDPR), were often used as an excuse to restrict access to essential information about children in TA:

*"Internally, we just say GDPR is a b******* excuse because we're talking about safeguarding of vulnerable kids…So, if this was a domestic violence issue and a kid had been beaten up, everyone would know but we put these really vulnerable families in incredibly vulnerable situations in B and Bs with other kind of difficult single adults, or we put them out of borough, or we put them in disgusting accommodation, and suddenly, no one's really bothered… all we want to do is go, tell us who they are and we'll help them, and they just say, Oh no, it's GDPR… if we're talking about safeguarding children, then you need to share the data with us." (3597)*

Participants felt that the consequence of this limitation in access to essential information meant staff often had to take on additional tasks outside their usual responsibilities to obtain the details needed to support families:

"So I then have to do the secondary work of emailing other colleagues to say can you check the data, can you check if you've got any information for the landlord. Other than that, other teams might have access to the land registry, that tells us the type of property and who's the owner, whether they're a leaseholder or sub tenant etc. But again, you need a license for that, and only specific teams have that." (2226)

Moreover, the described that existing data collection processes failed to capture key demographic details about children, limiting the ability to tailor support services effectively:

"They don't even acknowledge the age of children, or the ethnicity of the family on their figures. One, because it's not important to them. Because if it was, it would be being measured… So these things we, we often will say we know how many people are in the household, but don't know the ages of the children. Where is that information being, because if you don't know how many children are in that situation, you can't change it. In that sense, that way the five year old needs to be in school, and that three year old need universal 16 hours of childcare provision, you know, to make sure there's some socialisation going on." (1151)

A further barrier identified was the lack of notification when families are relocated which disrupts continuity of care. Participants were concerned that without proper alerts, existing data-sharing systems will fail to support effective interagency collaboration, increasing the risk that vulnerable families are overlooked:

"The biggest barrier there is that no one tells any sort of health professional, any sort of social care professional when a family has declared themself homeless. Yeah, they

For peer review only

> *will be placed in temporary accommodation and no one's doing any notification." (6631)*

The impact of these data-sharing constraints was described as far-reaching, as many teams describe still receiving referrals through chance conversations rather than structured communication channels:

> *"I did have a family that transferred into temporary accommodation and we didn't know via housing or anything like that. It's only because the family nurse partnership team in that area contacted us and said oh did you know, and we were like okay, we'll take them on then." (3058)*

Furthermore, participants reported that just as families are connected to support services, they are often relocated before they can fully benefit from them:

> *"They come in and they go out and by the time somebody finds them they're about to go out. And they might be in there for weeks on end of having no services. By the time, this is my colleagues, they've got the services up and running they're just about to move out so they're not there to receive all the lovely services." (1663)*

Participants were concerned ensuring continuity of care for these families largely depended on the dedication and goodwill of individual professionals rather than a reliable information sharing system:

> *"It just relies on the goodwill of the individual practitioner, because as I said during this pandemic I could have just not done anything. Or I could have done something that I thought was, in many ways, helpful to my clients. Yeah, so it's not something that I would have been kind of expected to, if I couldn't, I wouldn't have been expected to do, I didn't have to do it." (1988)*

Discussion

To our knowledge, this is one of only a few qualitative studies, set in a high income country, which examines cross-sector professionals' experiences of delivering integrated care to children under 5 living in TA during the COVID-19 pandemic. This study builds on existing research highlighting barriers to accessing care faced by families from professionals' perspectives (12). While the pandemic motivated innovative and opportunistic service integration to meet demands, it also exposed the fragility and inequity of existing systems, which lacked the infrastructure needed for sustainable collaboration. The findings of this study reveal a complex web of systemic challenges, including siloed team structures, inadequate communication, lack of specialised training, resource constraints, and restrictive institutional policies - the majority of which remain barriers today (37). This study highlights how these barriers operate across individual, team, work environment, and institutional levels.

A key individual-level barrier was the lack of formal training for professionals, with many feeling unprepared and relying on experiential rather than structured evidence-based learning. Similar to previous research (38,39), the absence of training resources increased staff stress and lowered confidence. Limited professional development confined staff to reactive crisis management, reducing capacity for sustainable support and skill-building. This reactive focus aligns with previous research, which found that services working with vulnerable populations

For peer review only

are frequently crisis-driven and tend to diminish once immediate risks subside (40). Embedding tailored training into routine practice is essential to improve service continuity and workforce preparedness (41).

At the team level, conflicting priorities and poor communication, particularly between health and housing services, were significant barriers to integrated care, often sidelining the child's needs. These challenges led to inefficiencies, delays, and fragmented services, consistent with previous findings on cross-sector collaboration (40,42). Professionals often took on extra coordination tasks to bridge communication gaps and prevent families from falling through service gaps, increasing their workload and detracting from direct service delivery - a response similarly noted in a study on healthcare for vulnerable populations (43). Staffing shortages further disrupted service continuity, hindered relationship-building, and weakened collaboration, echoing existing research highlighting the impacts of workforce instability (44). The pandemic further strained already understaffed services (45), leaving professionals in a constant state of "firefighting" and contributing to widespread burnout (46,47). Post-pandemic challenges persist, with housing providers frequently hiring at pace to fill urgent gaps, often prioritising speed over skills and experience (48). This reactive approach not only affects service quality but also makes it harder to foster the stable, long-term professional relationships needed for effective, child-centred care.

Within the work environment, workload pressures led to teams operating in silos, with poor collaboration creating inefficiencies and backlogs. This often resulted in delayed referrals and missed opportunities for children in TA to access vital health and support services. While siloed working across housing and social sectors supporting homeless populations is well-documented (18,49,50) this study also found significant fragmentation within and with healthcare teams, where greater collaboration could have led to better outcomes for children.

Systemic funding constraints, particularly limited investment in prevention, was noted as a barrier to integrated care, aligning with existing research (51–53). Children under 5 in TA, already at a disadvantage, feel the impact of this most acutely, with the pandemic further widening health inequalities (12). The lack of investment in prevention not only undermines children's long-term health outcomes but also places greater strain on already overstretched services (53). Differences in catchment areas and eligibility criteria among services posed further challenges to integrated care. These inconsistencies often lead to unnecessary duplication of services, as highlighted by the Institute for Government which found that overlapping responsibilities can result in inefficiencies (28). More critically, this study found that such misalignments frequently disrupt continuity of care, causing vulnerable families to fall through the gaps in siloed support systems.

One of the most significant barriers to integrated care was the restriction of data sharing between teams and thus the inability to identify and track access and outcomes overtime and geography. The lack of interoperable IT systems is a well-documented challenge for integrated homeless services, limiting cross-sector collaboration (54). A 2015 survey by the Health Service Journal found that over 60% of professionals believed that data protection regulations were hindering their efforts to integrate health and social care services (55). Our findings also reveal that homeless children are often excluded from data-sharing structures designed to safeguard vulnerable groups, highlighting how current systems overlook their needs - an issue made especially visible during the pandemic (56). Greater legislative flexibility is needed to enable secure, efficient information sharing (40), especially for children in their formative years, as their frequent relocations increase the risk of them slipping through the gaps in care

For peer review only - http://bmjopen.bmj.com/site/about/guidelines.xhtml

(57,58). Enabling local systems to link and share data, professionals can gain a more complete picture of the health and social needs of homeless children (59).

Strengths

A key strength of this study was the combination of diverse cross-sector perspectives, capturing insights from professionals across health, housing, and social care. The national scope offers broad applicability across England and strengthens the transferability of the findings beyond local contexts. The use of semi-structured interviews and detailed topic guide that was informed by larger team's expertise in the area provided consistency across participants while allowing in-depth exploration of individual experiences, gathering rich nuanced insights into barriers faced by professionals. The research fellow holds a PhD in sociology and is well trained in the use of qualitative research methods. She was closely supervised by lead researchers on the project with in-depth contextual and methodological expertise relevant to this research. Additionally, the use of co-coding and consensus discussions during analysis enhanced the reliability and rigour of the findings, reducing individual bias and ensuring consistency in theme identification (60). The research also aligns with current national and local priorities, contributing to the recent all-party parliamentary group call for evidence on temporary accommodation and supporting NHS integrated care boards efforts to address health inequalities and homelessness (61). Finally, the study was supported by a team with significant expertise in health and social care systems research, strengthening the rigour and interpretation of the findings.

Limitations

A limitation of this study is that recruitment was particularly challenging as the study took place during the COVID-19 pandemic, when there were significant service disruptions, staff redeployment, and heightened professional responsibilities. Therefore, availability of some staff was limited, which extended recruitment timeframe and localities of where professionals were recruited from - relying on opportunistic sampling rather than systemic representation of service provision across England. As a result, the study does not account for local variation in service provision or allow for comparative analysis between regions. However, data saturation was reached, indicating that key themes were consistently identified across interviews despite recruitment challenges. Lastly, the data was collected almost 3 years ago, however, the findings remain valuable for informing current and future practices, particularly as services continue to recover from the pandemic and many of the barriers remain relevant.

Recommendations

Strengthening integrated care for children in TA requires targeted cross-sector training to build understanding of population needs and different service roles, improving collaboration and delivery. In addition, a notification system, as proposed by the Shared Health Foundation (56), could alert services when families enter TA, helping prevent children from falling through gaps. In the short-term, investment in key workers, shown effective in the Supporting Families programme (62), would support continuity of care for families facing relocations. Ultimately, insights from this study highlight the urgent need for substantial capital investment in integrated recovery programmes and the adoption of a universal theory driven model of integrated care that prioritises cross-sector collaboration, early intervention, and preparedness for future crises.

Future research

For peer review only

Future research should focus on a longer term post-COVID study to reflect on pandemic learnings and further examine persistent barriers to cross-sector collaboration in supporting children in TA. Future research should also explore how geographical differences affect the delivery and accessibility of support for children in TA. Local variation in resources, services, and inter-agency coordination may significantly influence outcomes and should be examined through comparative, place-based analysis. Lastly, further research focusing on the experiences and outcomes of families themselves would provide valuable insights into the real-world impacts of systemic barriers and help shape more child-centred policies.

Conclusion

This study highlights the complex, multi-level barriers that professionals face when delivering integrated care to children under 5 in TA. From siloed working practices and limited training to staffing shortages and restrictive data-sharing policies, these challenges collectively hinder cross-sector collaboration and reflect long-standing issues in service delivery. The findings highlight the urgent need for systemic reforms, including improved cross-sector communication and training initiatives, flexible data-sharing frameworks, and greater investment in integrated recovery plans to support children post pandemic. Leveraging cross-sector expertise and fostering interdisciplinary collaboration can cultivate a more skilled, coordinated workforce, ensuring equitable access to quality care for children under 5 in TA.

References

1. K. Richard, M. and Shinn , M. (2024) ‘Chapter 28: Preventing and ending family homelessness’, in *Research Handbook on Homelessness*. EE Elgar, pp. 374–387.
2. Hart, A. (2025) Record child homelessness statistics, Shelter England. Available at: https://england.shelter.org.uk/what_we_do/updates_insights_and_impact/record_child_homelessness_and_soaring_rough_sleeping_figures#:~:text=Temporary%20accommodation%20at%20an%20all%2Dtime%20high&text=The%20new%20statistics%20reveal%20that,new%20record%20in%20two%20years (Accessed: 01 March 2025).
3. At least 354,000 people homeless in England today (2024) Shelter England. Available at: https://england.shelter.org.uk/media/press_release/at_least_354000_people_homeless_in_england_today_ (Accessed: 01 January 2025).
4. Svirydzenka, N., Lakhanpaul, M. and Williams, J. (2025) *In their own words: Children's experiences in temporary accommodation*, *Shelter Scotland*. Available at: https://scotland.shelter.org.uk/professional_resources/policy_library/in_their_own_words_childrens_experiences_of_temporary_accommodation
5. Homeless in a pandemic: 253,000 people are trapped in temporary accommodation (2020) Shelter England. Available at: https://england.shelter.org.uk/media/press_release/homeless_in_a_pandemic_253000_people_are_trapped_in_temporary_accommodation_#:~:text=Shelter%20investigation%20exposes%20daily%20struggle,highest%20figure%20for%2014%20years (Accessed: 01 January 2025).
6. Wilson, W. and Barton, C. (2023) Households in temporary accommodation (England) - House of Commons Library, UK Parliament. Available at: https://commonslibrary.parliament.uk/research-briefings/sn02110/ (Accessed: 01 January 2025).

7. Rosenthal, D.M. *et al.* (2020) 'Impacts of covid-19 on vulnerable children in temporary accommodation in the UK', The Lancet Public Health, 5(5). doi:10.1016/s2468-2667(20)30080-3.
8. Coughlin, C.G., Sandel, M. and Stewart, A.M. (2020) 'Homelessness, children, and covid-19: A looming crisis', Pediatrics, 146(2). doi:10.1542/peds.2020-1408.
9. Abrams, E.M. *et al.* (2022) 'The COVID-19 pandemic: Adverse effects on the social determinants of health in children and families', Annals of Allergy, Asthma & Immunology, 128(1), pp. 19–25. doi:https://doi.org/10.1016/j.anai.2021.10.022.
10. Tapper, J., Fazackerley, A. and Thorpe, V. (2025) 'The pandemic reinforced existing inequalities – it was a magnifying glass': How covid changed Britain, The Guardian. Available at: https://www.theguardian.com/world/2025/mar/09/the-pandemic-reinforced-existing-inequalities-it-was-a-magnifying-glass-how-covid-changed-britain (Accessed: 13 March 2025).
11. Development matters (2023) GOV.UK - Department for Education. Available at: https://www.gov.uk/government/publications/development-matters--2/development-matters (Accessed: 01 January 2025).
12. Rosenthal, D.M. *et al.* (2023) 'How does living in temporary accommodation and the COVID-19 pandemic impact under 5s' healthcare access and health outcomes? A qualitative study of key professionals in a socially and ethnically diverse and deprived area of London', International Journal of Environmental Research and Public Health, 20(2), p. 1300. doi:10.3390/ijerph20021300.
13. Filip, R. *et al.* (2022) 'Global challenges to public health care systems during the COVID-19 pandemic: A review of pandemic measures and Problems', Journal of Personalized Medicine, 12(8), p. 1295. doi:10.3390/jpm12081295.
14. How providers are working together across systems in response to COVID-19 (2022) Care Quality Commission. Available at: https://www.cqc.org.uk/publications/major-reports/how-providers-are-working-together-across-systems-response-covid-19 (Accessed: 01 January 2025).
15. Bell, L., Whelan, M. and Lycett, D. (2022) 'Role of an integrated care system during COVID-19 and beyond: A qualitative study with recommendations to inform future development', Integrated Healthcare Journal, 4(1). doi:10.1136/ihj-2021-000112.
16. Monitoring the impact of COVID-19 fortnightly homelessness briefing 6: focusing on emergency hotel accommodation (2020) Groundswell. Available at: https://groundswell.org.uk/wp-content/uploads/2020/07/COVID-19-Fortnightly-Briefing-6-FINAL.pdf (Accessed: 01 January 2025).
17. Doran, K.M. and Tinson, A. (2021) 'Homelessness and Public Health: Lessons Learned from the COVID-19 Pandemic ', European Journal of Homelessness, 15(3), pp. 49–62. Available at: https://www.feantsaresearch.org/public/user/Observatory/2021/EJH_15-3/Final/EJH_15-3_A3.pdf.
18. Daly, M., Ryu, S. and Polat, E. (2023) *Child-related policies in the first year of the COVID-19 pandemic in 40 countries*, *UNICEF Innocenti Global Office of Research and Foresight*. Available at: https://www.unicef.org/innocenti/documents/child-related-policies-first-year-covid-19-pandemic-40-countries (Accessed: 01 March 2025).
19. Mann, E. *et al.* (2024) 'A qualitative exploration of the impact of covid-19 on families with a child under 5 years-old in the borough of Newham, East London', *Discover Social Science and Health*, 4(1). doi:10.1007/s44155-024-00082-4.
20. McGuinness, K. (2023) Worse than covid - how the cost of living crisis is undermining our hardest hit neighbourhoods, Northumbria Police & Crime Commissioner. Available at: https://northumbria-pcc.gov.uk/v3/wp-content/uploads/2023/05/Worse-than-covid-

how-the-cost-of-living-crisis-is-undermining-our-hardest-hit-neighbourhoods-FINAL.pdf (Accessed: 01 January 2025).

21. Arrieta, T. (2022) 'Austerity in the United Kingdom and its legacy: Lessons from the covid-19 pandemic', *The Economic and Labour Relations Review*, 33(2), pp. 238–255. doi:10.1177/10353046221083051.
22. Butler, P. (2024) *The devastating impact covid and austerity had on children in England*, *The Guardian*. Available at: https://www.theguardian.com/society/2024/feb/19/the-devestating-impact-of-covid-and-austerity-on-children-in-england (Accessed: 01 January 2025).
23. Cross, S. *et al.* (2021) 'Housing, homelessness and children's Social Care: Towards an urgent research agenda', *The British Journal of Social Work*, 52(4), pp. 1988–2007. doi:10.1093/bjsw/bcab130.
24. Tu, Y. *et al.* (2024) 'Interventions to promote the health and well-being of children under 5s experiencing homelessness in high-income countries: A scoping review', *BMJ Open*, 14(1). doi:10.1136/bmjopen-2023-076492.
25. CHAMPIONS Project (2023). CHAMPIONS Policy Brief, University College London: London. Available at: https://drive.google.com/file/d/1LEYxCNfqnUbgoxnts5Pmvbu50OgvIckn/view
26. Wilson, S. *et al.* (2015) *Joining up public services around local, citizen needs: Perennial challenges and insights on how to tackle them*, *Institute for Government* . Available at: https://www.instituteforgovernment.org.uk/sites/default/files/publications/4564%20IFG%20-%20Joining%20up%20around%20local%20v11c.pdf.
27. Dyakova, M. *et al.* (2021) 'Placing health equity at the heart of the COVID-19 sustainable response and recovery: Building prosperous lives for all in Wales', *The Welsh Health Equity Status Report initiative (WHESRi)*. Available at:https://phwwhocc.co.uk/wp-content/uploads/2021/07/WHESRi-Covid-Report-Eng.pdf.
28. Smith, J.A. and Judd, J. (2020) 'Covid-19: Vulnerability and the power of privilege in a pandemic', *Health Promotion Journal of Australia*, 31(2), pp. 158–160. doi:10.1002/hpja.333.
29. *Champions Project*. Available at: https://www.championsproject.co.uk/ (Accessed: 01 March 2025).
30. Palinkas, L.A. *et al.* (2013) 'Purposeful sampling for qualitative data collection and analysis in Mixed Method Implementation Research', *Administration and Policy in Mental Health and Mental Health Services Research*, 42(5), pp. 533–544. doi:10.1007/s10488-013-0528-y.
31. Parker, C, Scott, S, and Geddes, A (2019) *Snowball Sampling*. SAGE Research Methods Foundations. Available at: https://methods.sagepub.com/foundations/snowball-sampling
32. Taylor, S.J., Bogdan, R. and DeVault, M.L. (2016) *Introduction to qualitative research methods: A guidebook and resource*. Hoboken, N.J: John Wiley & Sons, Inc.
33. Vasileiou, K. *et al.* (2018) 'Characterising and justifying sample size sufficiency in interview-based studies: Systematic Analysis of qualitative health research over a 15-year period', *BMC Medical Research Methodology*, 18(1). doi:10.1186/s12874-018-0594-7.
34. Zoom Video Communications Inc. Security Guide-Zoom Video Communications Inc. Available at: https://d24cgw3uvb9a9h.cloudfront.net/static/81625/doc/Zoom-Security-White-Paper.pdf (Accessed: 01 Sept 2021).

35. Braun, V., & Clarke, V. (2006). Using thematic analysis in psychology. Qualitative Research in Psychology, 3(2), 77-101.
36. QSR International Best Qualitative Data Analysis Software for Researchers. NVivo. QSR International. (accessed on 12 December 2023). Available at: https://www.qsrinternational.com/nvivo-qualitative-data-analysis-software/home?creative=605555104699&keyword=nvivo&matchtype=e&network=g&device=c&gclid=Cj0KCQiAyMKbBhD1ARIsANs7rEEmS573T9mQwlPzfwU-JWgQACMl4I8_lDGdjuOTUU3vyBYIgXXqO5saAvuKEALw_wcB.
37. Hurst , G., Teixeira, L. and Davies, N. (2025) A smarter approach to homelessness, Institute for Government. Available at: https://www.instituteforgovernment.org.uk/sites/default/files/2025-05/A-smarter-approach-to-homelessness.pdf.
38. McNeil, R. et al. (2013) 'Learning to account for the social determinants of Health Affecting Homeless Persons', Medical Education, 47(5), pp. 485–494. doi:10.1111/medu.12132.
39. Cené, C.W. et al. (2010) 'Community-based teaching about health disparities: Combining education, scholarship, and Community Service', Journal of General Internal Medicine, 25(S2), pp. 130–135. doi:10.1007/s11606-009-1214-3. (Accessed: 1st Jan 2025).
40. Klassman, K. et al. (2024) 'A rapid evidence assessment of barriers and strategies in service engagement when working with young people with complex needs', Children and Youth Services Review, 156, p. 107292. doi:10.1016/j.childyouth.2023.107292. (Accessed: 1st Jan 2025).
41. Davis, J. and Lovegrove, M. (no date) *Inclusion Health: Education and training for Health Professionals: Report*, *Allied Health Solutions Limited*. Available at: https://assets.publishing.service.gov.uk/media/5a80ef0040f0b62305b8df8c/NIHB_-_Inclusion_Health_education__Report.pdf (Accessed: 01 January 2025).
42. Black, E.B. et al. (2018) 'Homeless youth: Barriers and facilitators for service referrals', Evaluation and Program Planning, 68, pp. 7–12. doi:10.1016/j.evalprogplan.2018.02.009. (Accessed: 01 January 2025).
43. Pierce, P. et al. (2024) 'Effects of UK hostile environment policies on maternity care for refugees, asylum seekers, and undocumented migrants in Camden: Examining the experiences of healthcare professionals and community organisations', Journal of Migration and Health, 11, p. 100291. doi:10.1016/j.jmh.2024.100291.
44. Phillips, A., Bush, L. and Walcott, S. (2023) Wall to wall support joining up public services and housing for vulnerable children, young people and families. Report. Available at: https://www.grosvenor.com/getattachment/f3ed168a-0fbd-4e83-ab80-f55d6bd67e95/Wall-to-Wall-Support-final-version-EMBARGOED-UNTIL-07-12-23.pdf (Accessed: 01 January 2025).
45. Ofsted (2022) Ofsted: Concerns over damaging impact of staff shortages on children's Social Care, GOV.UK. Available at: https://www.gov.uk/government/news/ofsted-concerns-over-damaging-impact-of-staff-shortages-on-childrens-social-care#:~:text=The%20report%20finds%20that%20the,staff%20are%20able%20to%20support (Accessed: 01 January 2025).
46. Frontline Network (2023) Experiences of frontline homelessness work 2022. rep. Available at: https://www.frontlinenetwork.org.uk/media/2794/frontlinenetworksurvey-workforcefindings-1603.pdf (Accessed: 01 January 2025).
47. Flores, E.V. (2023) Organizational strategies to improve retention among the homeless sector workforce: A Systematic Literature Review. California State University, Long

Beach ProQuest Dissertations & Theses. Available at: https://www.proquest.com/openview/525a631b9ed30aeb9372088a417d83df/1?cbl=18750&diss=y&pq-origsite=gscholar

48. The National Housing Federation (2024) The Better Social Housing Review. Report. Available at: https://s46834.pcdn.co/wp-content/uploads/BSHR_Report_FINAL_embargoed_until_Tues13thDec.pdf
49. Cornes, M. , Joly, L. , Manthorpe, J. and O'Halloran, S. (2011), *Rethinking Multiple Exclusion Homelessness: Implications for Workforce Development and Interprofessional Practice (Summary of Findings)*, The King's College London, London. Available at: https://insight.cumbria.ac.uk/id/eprint/4171/1/OHalloran_RethinkingMultiple.pdf
50. Morgan Diamond, M. (2020) 'Pathway to Opportunity: Apprenticeships and Breaking the Cycles of Poverty for Young People Experiencing Homelessness', Young Invincibles. Available at: https://younginvincibles.org/wp-content/uploads/2020/08/IL_Apprenticeship_Pathway_Final_2-min.pdf
51. Hughes, G., Shaw, S.E., Greenhalgh, T. (2020) 'Rethinking integrated care: A systematic hermeneutic review of the literature on Integrated Care Strategies and concepts', The Milbank Quarterly, 98(2), pp. 446–492. doi:10.1111/1468-0009.12459.
52. Wood, C., Salter, J. and Cheetham, P. (2012) Under one roof. rep. Demos. Available at: https://demos.co.uk/wp-content/uploads/2012/05/Under_one_roof_-_web.pdf (Accessed: 01 January 2025).
53. Curtis, P., Glover, B. and O'Brien, A. (2023) The preventative state rebuilding ur local, social and civic foundations. rep. Demos. Available at: https://demos.co.uk/research/the-preventative-state-rebuilding-our-local-social-and-civic-foundations/ (Accessed: 01 January 2025).
54. Ní Cheallaigh, C. (2021) 'Barriers and facilitators of an integrated, Interdisciplinary Inclusion Health Service in Dublin, Ireland.', International Journal of Integrated Care, 21(S1), p. 112. doi:10.5334/ijic.icic20463.
55. Dalton, R. (2017) Integration: Local government and health leaders prefer a whole system approach, Health Service Journal. Available at: https://www.hsj.co.uk/supplement-archive/integration-local-government-and-health-leaders-prefer-a-whole-system-approach/5086572.article#.VhKmJvlVhBc (Accessed: 01 January 2025).
56. Families experiencing homelessness: Notification system (2020) Shared Health Foundation. Available at: https://sharedhealthfoundation.org.uk/uncategorized/families-experiencing-homelessness-notification-system/ (Accessed: 01 January 2025).
57. Russell, M. and Lister, R. (2020) Moving Always Moving. rep. The Children's Society. Available at: https://www.childrenssociety.org.uk/sites/default/files/2020-10/Moving-Always-Moving-Report.pdf (Accessed: 01 January 2025).
58. Mettle-Olympio, B. (2024) Temporary accommodation in London: Causes, consequences, and solutions, Capital Letters. Available at: https://capitalletters.org.uk/temporary-accommodation-in-london-causes-consequences-and-solutions/#:~:text=Children%20living%20in%20temporary%20accommodation%20are%20more%20likely%20to%20experience,stress%20of%20their%20living%20situation (Accessed: 01 January 2025).
59. Caswell, R. (2021) Homelessness and the response to COVID-19: learning from lockdown Data and data sharing, Strategy Unit NHS. Available at: https://www.strategyunitwm.nhs.uk/sites/default/files/2021-

02/Homelessness%20data%20and%20data%20sharing%20Strategy%20Unit%20Final%20Report.pdf (Accessed: 01 January 2025).

60. Cole, R. (2023). Inter-Rater Reliability Methods in Qualitative Case Study Research. Sociological Methods & Research, 53(4), 1944-1975. https://doi.org/10.1177/00491241231156971

61. Procter, A., Pratt, S. and Wise-Martin, I. (2023) Call for evidence findings: Summary, analysis of themes and call to action, *APPG for Households in Temporary Accommodation*. Available at: https://householdsintemporaryaccommodation.co.uk/reports/call-for-evidence-findings-summary-analysis-of-themes-and-call-to-action/ (Accessed: 01 March 2025).

62. MacAlister, J. (2021) The Case for Change. rep. The independent review of children's social care. Available at: https://fdac.org.uk/wp-content/uploads/2021/06/case-for-change.pdf (Accessed: 01 January 2025).

For peer review only

Professional Group	Number of Professionals n (%)
Health Visitors	20 (9)
Health Professionals	35.6 (16)
Non-Profit Sector	22.2 (10)
Housing Sector	17.8 (8)
Education	4.4 (2)
**Age Group**	**n (%)**
Not given	4.4 (2)
25–29	6.7 (3)
30–34	15.6 (7)
35–39	13.3 (6)
40–44	15.6 (7)
45–49	22.2 (10)
50–54	2.2 (1)
55–59	6.7 (3)
60–64	11.1 (5)
65-69	2.2 (1)
**Gender**	**n (%)**
Not given	2.2 (1)
Male	11.1 (5)
Female	83.7 (39)
**Ethnic Group**	**n (%)**
Not given	6.7 (3)
White (British and/or Any other White British background)	77.8 (35)
Black (African or Caribbean)	6.7 (3)
Asian or Asian British: Indian	6.7 (3)
Mixed Ethnic background	2.2 (1)
**How Long Professionals Have Worked with Families Living in Temporary Accommodation (Time Spans)**	**n (%)**
<1 year	0
1-5 years	26.7 (12)
5-10 years	33.3 (15)
>10 years	40 (18)
**Total**	**45 (100%)**

Themes	Sub-themes
Individual factors	Lack of training and knowledge
	Individual constraints to upskill
Team factors	Misaligned goals between teams
	Poor communication between teams
Work environmental factors	Reduced staffing levels and high staff turnover
	Workload and capacity issues
Institutional context factors	Lack of funding and senior support
	Policy constraints and catchment area limitations
	Lack of specialised services
	Data sharing and access issues

For peer review only

CHAMPIONS Interview schedule, Professionals v0-6

Opening

- Personal introduction to researcher
- Interview topics to be covered and focus of interview – ie what is different about living in COVID times compared to living in TA before this
- Interview length, format and breaks
- Confidentiality and consent
- Any questions

Scene-setting

Please tell me a little bit about your role:

- What services do you provide to families experiencing homelessness? Is this service specifically for families or also for other groups?
- Does your area have any services specifically for children?
- How do you work with them under normal circumstances?
- How much experience do you have in working with families living in TA? How do you find out about the specific set of problems these children might face?
- What training do staff have received that is relevant to the group they work with. [If they identify anything] Is this provided in house or externally? Did you source it yourself or is it offered by your employer?
- Are you or other members of your team trained or skilled specifically to work with a) children, b) children in TA?
- Are you familiar with your families' accommodation? Do you have any comments about the quality and safety of the environment?

Focus on specific topics

[Follow up questions will be asked to further explore the key topics of interest where they are not covered already by the discussion of photos]

1. Contact and links with other services

Is families' or the child's homelessness status relevant to the service you provide? If so, what do you do differently with them that you don't do with other families, children or client groups?

Can you tell me a bit about how children in your area come to light to the different services and which services? Which organisation or person is responsible for looking after families / children living in TA? Who keeps track of families? What happens if families move?

Are children in TA referred to you: eg is it flagged on an IT system; are you informed, or do you need to ask?

What are the barriers to finding out about their homelessness status and providing appropriate services? (knowledge, skills, access to data, communication internally or with other agencies)

Are there other barriers to providing a service to this group of children and their families? Do you know of any barriers that families experience in accessing your service?

Is your service part of a pathway for families/children to access your service? Who refers them? Do you refer them on to other agencies, and which ones? Do you work in tandem, and how effective is

For peer review only - http://bmjopen.bmj.com/site/about/guidelines.xhtml

that? Who follows up to ensure that families continue to access services?

How do these various services link together?

2. COVID Knowledge:

How does your organisation find out about COVID restrictions, and keep up to date with changes? How useful were the sources of information and how well did they apply to your service and service users? Who makes decisions about how to define and when to use safe working practices? How are staff informed about this?

Are you or your team responsible for cascading information about COVID restrictions to families, and how do you go about that? How well has that worked? What worked/what didn't?

3. How has COVID changed the way you work?

We began by asking how you normally go about providing your service.

To what extent do you know, based on your experience, how different are the challenges under covid? How has this changed in terms of:

- Access to families or children to provide your service and the length of time it takes
- Access and referrals to other services such as health visitors, medical care, development checks, dental care, ongoing care for physical/mental health issues
- The way you assess their needs.
- Has the assessment method changed?
    - How have families/children's needs changed since covid? How has your service responded to that?
- Are any of these changes likely to become 'business as usual' for your service delivery?
- Has there been a difference in service delivery between full lockdown and living with COVID restrictions?
- Have you been able to access any additional funding or resources (e.g. staff) to support the service change needed to deal with the pandemic? If so how did that come about, was it a request or it came from (local) government? Do you think it will be sustained beyond 2021?

Do you have any recommendations for what could be done to address these issues?

4. What impact is COVID having on families living in TA? → with examples if possible

From your knowledge of the families, what do you think their biggest concerns have been?

Have these issues affected their ability to engage with [your service]?

What do you think has been the greatest impact on children under 5 living in TA through the COVID pandemic and how has it affected their [health/wellbeing and development/education]? What have your main worries / concerns/observations been?

- Child development (health and social aspects)
- Housing quality/issues and access to (safe) outdoor space/play areas
- Nutrition: cooking and eating
- Education
- Wellbeing/Resilience in general

For peer review only - http://bmjopen.bmj.com/site/about/guidelines.xhtml

- Factors that complicate the situation or make it more difficult, such as disability

Are you aware of the local TA boards within their own LA or borough which discuss how to set up and manage services for homeless families? Is there one locally? Is your service involved with it?

Health visitors:

Thinking about the targets set in the Healthy Child Programme, do you think the pandemic has affected the child's:

- mental health
- physical development
- emotional wellbeing
- diet and access to healthy food

and how? What are the priority areas for recovery?

What support do families need to ensure this happens, and which organisations do you need to work with to provide it?

Health professionals:

Charities:

How much of your service provision relies on fundraising, and has COVID affected this?

Has there been any discussion of the financial and wider sustainability of services? Have any decisions been made about changing the way the service is provided?

Housing teams:

How have you found out about the specific needs of people who use your service?

Have your families needed any additional support from your service, such as

- Signposting or support to access welfare services
- Food banks
- Mental health support

What happens to the child or family after they move on from your service?

What happens if the TA accommodation allocated to a family with children is unsuitable for their needs, for example is overcrowded, has dampness/mould, is in need of repairs, has access issues for prams/buggies, is shared with other unsuitable residents: how would you become aware of these problems (e.g. families complain, or regular property inspections) and what are the options available to you for re-housing them and/or making repairs if appropriate?

Education:

Do you know which pupils are living in TA? Have you identified any specific needs for this group?

Are there any services or approaches that you take to support them?

(access to outdoors and spaces to play)

How has COVID 19 affected the education and school-readiness of this specific group? Does the

For peer review only - http://bmjopen.bmj.com/site/about/guidelines.xhtml

school provide additional support (one to one sessions, food parcels, uniform bank, access to IT, printed work pack etc)

Have they been able to access online learning – and what are the barriers to that?

What support do children need during pandemic recovery and afterwards?

Potential solutions

We have discussed your service runs, and how it supports families through the system, and how that has changed during COVID. I want to ask your views on how we can provide a better service for the future, and we set up a system that supports these children.

1. Earlier in our conversation you identified some major issues [such as...] for professionals in your field who are supporting families. What action do you think would address these concerns?
2. We have seen many new innovations and strategies during COVID such as the vaccination bus, the vaccination camps etc. How can we reach families who sometimes find it hard to access services ie how do we bring them services they need closer to them?
3. We are hearing stories about children being 'left behind' following the COVID restrictions, for example low weight, delayed language development and immunisations, and early signs of mental health problems, and there's a view we will need to help children catch up or address problems arising from COVID restrictions. What do you think we should be doing for these children to help them catch up and overcome some of the problems they have gone through?
4. At a national level:
   a. what do you think we should be advocating for to support children - what should our 'ask' be from health, housing, education, local government and central government?
   b. What are the top three actions, or services/facilities that need to be put in place, that you would like to see?
   c. What are your views on setting up an integrated care package around each child?
   d. What are your views on setting up a national notification system?
   e. Who are the key stakeholders in this process?

Closing

Is there anything else you would like to add that we haven't discussed so far?

If you'd like to add any further thoughts or comments, then please do so by contacting me at the email address or postal address on the information sheet.

At the end of the project we will write a summary of the results and send you a copy if you wish.

Thank you for taking part in this research.

For peer review only - http://bmjopen.bmj.com/site/about/guidelines.xhtml

COREQ (COnsolidated criteria for REporting Qualitative research) Checklist

A checklist of items that should be included in reports of qualitative research. You must report the page number in your manuscript where you consider each of the items listed in this checklist. If you have not included this information, either revise your manuscript accordingly before submitting or note N/A.

Topic	Item No.	Guide Questions/Description
Interviewer/facilitator	1	Which author/s conducted the interview or focus group? **KF**
Credentials	2	What were the researcher's credentials? E.g. PhD, MD **MA, PhD**
Occupation	3	What was their occupation at the time of the study? **Research Fellow**
Gender	4	Was the researcher male or female? **Female**
Experience and training	5	What experience or training did the researcher have? **MA in Social Research, 2 years of conducting qualitative market research interviews, PhD in Sociology**
Relationship established	6	Was a relationship established prior to study commencement? **No**
Participant knowledge of the interviewer	7	What did the participants know about the researcher? e.g. personal goals, reasons for doing the research **Participants were introduced to the researcher as a person working on a project with larger goals relating to identifying children's needs post-covid, co-developing requirements for covid recovery and reporting them to policymakers and practitioners.**
Interviewer characteristics	8	What characteristics were reported about the interviewer/facilitator? e.g. Bias, assumptions, reasons and interests in the research topic
Methodological orientation and Theory	9	What methodological orientation was stated to underpin the study? e.g.grounded theory, discourse analysis, ethnography, phenomenology, content analysis **Initial descriptive coding and thematic analysis.**
Sampling	10	How were participants selected? e.g. purposive, convenience, consecutive, snowball **Purposive, convenience and snowball sampling.**
Method of approach	11	How were participants approached? e.g. face-to-face, telephone, mail, Email<br>• **Via housing, welfare, refugee and DV support organisations and housing providers (LAs, HAs)**<br>• **Direct requests from professionals eg health visitor, housing officer**<br>• **Social media and WhatsApp, both owned by the project team and shared by support organisations**<br>• **Leaflets and posters shared by food banks, family support charities**
Sample size	12	How many participants were in the study? **45**
Non-participation	13	How many people refused to participate or dropped out? Reasons?
Setting of data collection	14	Where was the data collected? e.g. home, clinic, workplace **Online at a place of the participants' choice – usually their workplace or home office.**
Presence of non-participants	15	Was anyone else present besides the participants and researchers? **No. Two joint interviews took place with two colleagues.**
Description of sample	16	What are the important characteristics of the sample? e.g. demographic data, date **demographic data: role, age, gender, ethnicity, and length of time in role.**
Interview guide	17	Were questions, prompts, guides provided by the authors? Was it pilot-tested? **A brief agenda was sent before the interview outlining the topics to be covered and reassuring participants they did not have to answer any questions they were uncomfortable with. The topic guide was pilot tested with xx participants then reviewed by the research team.**
Repeat interviews	18	Were repeat inter views carried out? If yes, how many? **No.**
Audio/visual recording	19	Did the research use audio or visual recording to collect the data? **The sessions were audio-recorded, then transcribed and anonymised.**
Field notes	20	Were field notes made during and/or after the interview or focus group? **No.**

For peer review only

Duration	21	What was the duration of the interviews or focus group? **Interviews were scheduled to take no more than an hour but were shorter or longer according to the participants' time available and their convenience.**
Data saturation	22	Was data saturation discussed?
Transcripts returned	23	Were transcripts returned to participants for comment and/or correction?

Topic	Item No.	Guide Questions/Description
Number of data coders	24	How many data coders coded the data? **Two**
Description of the coding tree	25	Did authors provide a description of the coding tree?
Derivation of themes	26	Were themes identified in advance or derived from the data? **Both. The coding tree was drafted from the interview topic guide and additional codes were derived from the data.**
Software	27	What software, if applicable, was used to manage the data? **NVivo**
Participant checking	28	Did participants provide feedback on the findings?
Quotations presented	29	Were participant quotations presented to illustrate the themes/findings? Was each quotation identified? e.g. participant number
Data and findings consistent	30	Was there consistency between the data presented and the findings?
Clarity of major themes	31	Were major themes clearly presented in the findings?
Clarity of minor themes	32	Is there a description of diverse cases or discussion of minor themes?

Developed from: Tong A, Sainsbury P, Craig J. Consolidated criteria for reporting qualitative research (COREQ): a 32-item checklist for interviews and focus groups. *International Journal for Quality in Health Care*. 2007. Volume 19, Number 6: pp. 349 – 357

Once you have completed this checklist, please save a copy and upload it as part of your submission. DO NOT include this checklist as part of the main manuscript document. It must be uploaded as a separate file.

For peer review only

BMJ Open

Exploring barriers to integrated care for children under five living in temporary accommodation: a qualitative study of professionals’ experiences during the COVID-19 pandemic in England

Journal:	*BMJ Open*
Manuscript ID	bmjopen-2025-106318.R1
Article Type:	Original research
Date Submitted by the Author:	11-Jan-2026
Complete List of Authors:	Pierce, Poppy; University College London Faculty of Medical Sciences, Medical School<br>Svirydzenka, Nadzeya; De Montfort University Faculty of Health and Life Sciences, Applied Social Sciences<br>Adil, Haleema; University College London Faculty of Medical Sciences, Medical School<br>Allaham, Shereen ; UCL GOS ICH, Population, Policy and Practice Research and Teaching Department<br>Ankers, Matthew; Flinders University College of Nursing and Health Sciences,<br>Parry, Yvonne; Flinders University, School of Nursing & Midwifery<br>Heys, Michelle; UCL GOS ICH, Population, Policy and Practice Research and Teaching Department; East London NHS Foundation Trust, Specialist Children’s and Young People’s Services<br>Ucci, Marcella; University College London, Bartlett School of Graduate Studies<br>Lakhanpaul, Monica; UCL GOS ICH, Population, Policy and Practice Research and Teaching Department; Nottingham University Hospitals NHS Trust, Community Paediatrics
**Primary Subject Heading**:	Public health
Secondary Subject Heading:	Paediatrics
Keywords:	Delivery of Health Care, Integrated, Family, COVID-19, Health Services, Community child health < PAEDIATRICS, Vulnerable Populations

SCHOLARONE™
Manuscripts

I, the Submitting Author has the right to grant and does grant on behalf of all authors of the Work (as defined in the below author licence), an exclusive licence and/or a non-exclusive licence for contributions from authors who are: i) UK Crown employees; ii) where BMJ has agreed a CC-BY licence shall apply, and/or iii) in accordance with the terms applicable for US Federal Government officers or employees acting as part of their official duties; on a worldwide, perpetual, irrevocable, royalty-free basis to BMJ Publishing Group Ltd ("BMJ") its licensees and where the relevant Journal is co-owned by BMJ to the co-owners of the Journal, to publish the Work in this journal and any other BMJ products and to exploit all rights, as set out in our licence.

The Submitting Author accepts and understands that any supply made under these terms is made by BMJ to the Submitting Author unless you are acting as an employee on behalf of your employer or a postgraduate student of an affiliated institution which is paying any applicable article publishing charge ("APC") for Open Access articles. Where the Submitting Author wishes to make the Work available on an Open Access basis (and intends to pay the relevant APC), the terms of reuse of such Open Access shall be governed by a Creative Commons licence – details of these licences and which Creative Commons licence will apply to this Work are set out in our licence referred to above.

Other than as permitted in any relevant BMJ Author's Self Archiving Policies, I confirm this Work has not been accepted for publication elsewhere, is not being considered for publication elsewhere and does not duplicate material already published. I confirm all authors consent to publication of this Work and authorise the granting of this licence.

For peer review only

Exploring barriers to integrated care for children under five living in temporary accommodation: a qualitative study of professionals' experiences during the COVID-19 pandemic in England

Authors and affiliations

Poppy Pierce* - Medical School, Faculty of Medical Sciences, University College London, London, United Kingdom poppy.pierce.19@ucl.ac.uk

Nadzeya Svirydzenka* - School of Applied Social Sciences, Faculty of Health and Life Sciences, De Montfort University, Leicester, United Kingdom nadzeya.svirydzenka@dmu.ac.uk

Haleema Adil - Medical School, Faculty of Medical Sciences, University College London, London, United Kingdom haleema.adil.19@ucl.ac.uk

Shereen Allaham - Population, Policy and Practice Research and Teaching Department, University College London Great Ormond Street Institute of Child Health, London, United Kingdom s.laham@ucl.ac.uk

Matthew Ankers - College of Nursing and Health Sciences, Flinders University, South Australia matthew.ankers@flinders.edu.au

Yvonne Parry - Caring Futures Institute, Flinders University, South Australia yvonne.parry@flinders.edu.au

Michelle Heys - Population, Policy and Practice Research and Teaching Department, University College London Great Ormond Street Institute of Child Health, London, United Kingdom m.heys@ucl.ac.uk

- Specialist Children's and Young People's Services, Newham, East London NHS Foundation Trust

Marcella Ucci - Institute for Environmental Design and Engineering, Bartlett Faculty of the Built Environment, University College London, London, United Kingdom m.ucci@ucl.ac.uk

Monica Lakhanpaul** - Population, Policy and Practice Research and Teaching Department, University College London Great Ormond Street Institute of Child Health, London, United Kingdom m.lakhanpaul@ucl.ac.uk

- Community Paediatrics, Nottingham University Hospitals NHS Trust

*Poppy Pierce and Nadzeya Svirydzenka are joint first authors
**Monica Lakhanpaul is the senior author

Correspondence to:

Monica Lakhanpaul
m.lakhanpaul@ucl.ac.uk

Keywords: Integrated systems; family homelessness; child homelessness; temporary accommodation; COVID-19; policy and practice

For peer review only

ABSTRACT

Objectives: This research aims to explore the factors that hinder professionals in delivering integrated care for children under 5 in temporary accommodation (TA) and understand their experiences of collaboration during the pandemic to inform recommendations.
Design: Semi-structured qualitative interviews.
Setting: England, United Kingdom.
Participants: 45 professionals working across health, housing, education, and non-profit sectors in England. Purposive and snowball sampling was employed to recruit a representation of key professionals across England. Those not eligible to take part in the study included people who did not work with families and/or children in TA settings.
Outcome measures: To explore cross-sector learnings that are applicable to improving integrated care and to tailor recommendations to the needs of families and children under 5 experiencing homelessness in the UK today.
Results: This study highlights the complex, multi-level barriers that professionals face when delivering integrated care to children under 5 in TA. Findings are organised using a framework that distinguishes between practice-level, organisational, and systemic challenges. From siloed working practices and limited training to staffing shortages and restrictive data-sharing policies, these challenges collectively hinder service continuity and collaboration.
Conclusions: Although this project was conducted during the COVID-19 pandemic, the challenges identified reflect deeper, long-standing issues in service delivery. As services continue to recover and prepare for future crises, these insights remain highly relevant and can inform more resilient, integrated recovery plans to support children in TA beyond the pandemic context. Addressing these barriers, through improved collaboration, training, and data-sharing, is key to strengthening care for this vulnerable population.

Strengths and limitations of this study

- The study included diverse professionals from health, housing, and social care across England, enhancing relevance and transferability.
- Semi-structured interviews were conducted with a detailed topic guide, allowing for consistency and rich, nuanced data collection.
- Limited staff availability due to the COVID-19 pandemic led to opportunistic rather than representative sampling.
- The study could not account for local variation in services or enable comparative regional analysis.

INTRODUCTION

Family homelessness is an escalating issue within high-income countries, including the United States and across Europe [1]. In the UK, this trend is particularly alarming, with over 164,000 children living in temporary accommodation (TA) with their families in 2024; an increase of 15% since 2023 [2]. The rising numbers reflect the deepening housing crisis, exacerbated by economic pressures, a lack of liveable housing stock, and inadequate access to affordable housing [3]. The 2025 Shelter Scotland report provides stark evidence of how living in TA harms children’s health, development, and education, reinforcing the urgent need for cross-sector action to prevent long-term consequences [4]. The COVID-19 pandemic saw a sharp increase in the number of families living in TA across England, impacting an estimated 253,000 individuals [5,6]. Children under 5 were particularly susceptible to poor health outcomes, due to limited healthcare access; unstable and substandard living conditions; and the disruption to early development [7-9]. The COVID-19 pandemic inadvertently served as a stress test for

For peer review only

public services, showcasing effective practices but also exposing pressing issues within these systems [10]. Addressing these enduring challenges is urgent, as this is a critical period of rapid growth for children under 5, where timely intervention can prevent long-term developmental challenges and offer a unique opportunity to positively shape a child's futures [7,11,12].

The COVID-19 pandemic significantly disrupted care systems worldwide but also compelled health and social care partners to collaborate more closely and rapidly than they had previously [13,14]. Professionals reported improved cross-organisational working as priorities aligned to address the crisis [15]. Integrated community health responses and initiatives such as 'Everyone In' helped maintain care access and positioned homelessness as a public health priority [16,17]. However, children were largely overlooked in COVID-19 policy and planning, with low-income families with children under 5 being disproportionately disadvantaged [18,19]. Long-standing research has consistently identified structural barriers to effective health, housing, and social care provision for homeless families, well before the COVID-19 pandemic. Studies spanning the past two decades highlight persistent challenges including fragmented service delivery, limited cross-sector coordination, workforce instability, and bureaucracies across systems supporting both adults and children experiencing homelessness [20-23]. The pandemic highlighted and intensified numerous of these pre-existing vulnerabilities in the systems that support children under 5 in TA, such as staff shortages, limited training, and communication breakdown between teams [12]. Following the pandemic, many of these issues remain unresolved, with additional challenges like the cost of living, austerity measures, and the housing crisis continuing to hinder recovery [24-27]. The result is overlapping crises which are also likely to be exacerbating each other. As we anticipate future crises, it is imperative to address healthcare provision for children under 5 in TA and how to sustain such provision in a system under stress. The majority of children under 5 in TA face complex and multifaceted challenges, where solutions do not necessarily lie within the remit of a singular organisation [28,29]. Effective cross-sectoral action is needed to meet current demands, and the work to recover, prepare, and innovate must be framed through a health equity lens [29-32].

While previous research has extensively documented systemic barriers to integrated care for homeless populations, less is known about how these long-standing challenges were experienced, adapted to, or exacerbated during the COVID-19 pandemic. Although research on children under 5 in TA during the pandemic is scarce, one study conducted in Newham, an exceptionally deprived and diverse inner-city London borough, offers valuable local insight into professionals' perspectives on the barriers families in TA faced in accessing healthcare during this time [12]. While the previous study focused on professionals' perspectives on families' challenges within a single borough, this study shifts the lens to professionals' reflections on their own roles and the barriers to cross-sector collaboration at a national level. In doing so, it builds on and extends existing knowledge, addressing a critical gap in understanding how to improve cross-sector working to better support children under 5 in TA. This research aims to qualitatively explore the factors that hinder professionals in delivering integrated care for children under 5 in TA and understand their experiences of collaboration during the pandemic to inform recommendations. The recommendations follow two key objectives: (i) to explore cross-sector learnings that are applicable to improving integrated care; (ii) tailor recommendations to the needs of families and children under 5 experiencing homelessness in the UK today.

METHODS

For peer review only

This study sits within the CHAMPIONS programme of research which focuses on the impact of living in poverty on children under 5 living in TA [33]. During the COVID-19 pandemic, a mixed method multi-phased study was conducted with the specific aim of understanding the experiences of children in TA during that time. This paper represents only the work package dedicated to the qualitative interviews with professionals engaged with and supporting the cross system around the child and their families.

Participants

Purposive and snowball sampling was employed [34,35] to recruit a representation of key professionals across England. Those not eligible to take part in the study included people who did not work with families and/or children in TA settings. No participants were excluded based on protected characteristics or any demographic factors. Participants were recruited through a variety of channels, including:

- Housing, welfare, refugee, and domestic violence support organisations, as well as housing providers such as local authorities and housing associations.
- Direct referrals from professionals, including health visitors and housing officers.
- Social media platforms and WhatsApp groups managed by the project team and partner support organisations.
- Leaflets and posters distributed through food banks and family support charities.
- Snowballing through word of mouth.

Recruitment was undertaken between September 2021 and March 2022. Participants were identified professionals within key areas who were invited to take part in the study via an email, leaflet, or word of mouth. All potential participants were asked to email the researchers to indicate their interest in taking part. Interested participants received an information sheet and were encouraged to respond with any questions, or to arrange an interview. Informed consent was obtained prior to participation. Research fellow on the project continued with recruitment and data collection until thematic saturation was reached, which occurred after 45 interviews [36]. Despite the challenges of conducting research during the COVID-19 pandemic, the study achieved a strong sample size for qualitative research as data saturation was reached [37]. Effective recruitment was facilitated by strong participant engagement strategies, established trust, and the research team's experience and credibility in the field.

Materials

The interview schedule was developed by looking into key domains of supporting families with children under 5 in TA. To gather detailed insights into the perceived barriers to delivering integrated care during the pandemic, the questions addressed topics such as training related to supporting children under 5 in TA; workload and capacity; workplace dynamics and collaboration between sectors, particularly between health, non-profit, and housing services; impact of COVID-19 pandemic on families living in TA; the impact of policies and funding on service delivery; personal approaches to providing care; and recommendations for improving integrated care for children under 5 in TA. The topic guide is presented in Supplementary File 1. A demographic questionnaire was also given to participants to complete prior to the interviews. A brief agenda was sent before the interview outlining the topics to be covered and reassuring participants that they did not have to answer any questions they were uncomfortable with.

For peer review only - http://bmjopen.bmj.com/site/about/guidelines.xhtml

KF arranged individual semi-structured interviews with participants, conducted remotely via Zoom [38] at a place of the participants' choice, usually their workplace or home office. KF is a research fellow, with a background of a PhD in sociology, MA in social research, MPhil, BA (Hons), with significant experience in conducting qualitative market research interviews. This was disclosed to participants. A relationship with participants and KF was not established prior to the interviews taking place. Participants were introduced to the researcher as a person working on a project with larger goals relating to identifying children's needs post-covid, co-developing requirements for covid recovery and reporting them to policymakers and practitioners. All interviews were carried out in English, recorded using Zoom's digital platform. Interviews were scheduled to take no more than an hour but were shorter or longer according to the participants' time available and their convenience. Only the researcher and participant were present at the interview. No repeat interviews were carried out and no field notes were made. Transcription was carried out by the research fellow or professional transcriber and quality cross checked with the audio file.

The COREQ reporting checklist is provided in Supplementary File 2.

Ethics approval and participant informed consent

Ethics approval was obtained by the UCL Research Ethics Committee (ID number: 9277.004). Verbal and written informed consent were obtained prior to participation. Participants were informed that their involvement was entirely voluntary.

Analysis

All interview transcripts were anonymised and given a four-digit number code. Researchers PP and HA carried out data analysis using an inductive approach to generate codes through data familiarisation. They were supported throughout by senior team members, including a paediatrician with in-depth knowledge of health and social care systems and academic researchers with expertise in qualitative methods and the relevant subject matter, which strengthened the rigour and interpretation of the findings. Two interviews were co-coded independently and codes were then compared to ensure replicability and reliability. In cases where conflict in codes arose, consensus discussions took place and codes were revised. In the final stage, findings were organised using a multi-level framework that distinguishes between practice-level, organisational, and systemic challenges. Codes were subsequently categorised into sub-themes that emerged in analysis. This followed Braun and Clarke's 6-step framework for qualitative analysis [39]. NVivo software (v.12) was used to assist in the organisation and analysis of the data [40].

Patient and public involvement

None.

RESULTS

In total, 45 interviews were conducted. The average time for an interview was 1 hour. Table 1 shows participant characteristics. Professionals included 9 Health Visitors, 16 Health Professionals, 10 professionals from Non-Profit organisations, 8 Housing Sector professionals and 2 professionals working in education. The participants' ages ranged from 26 to 68 years, with a mean age of 44. The majority were female (39/45), and they had experience working

For peer review only

with families living in TA for periods ranging from 1 to over 40 years, with an average of 8 years.

Table 1. Participant characteristics including role, age, gender, ethnicity, and length of time in role.

Professional Group	Number of Professionals n (%)
Health Visitors	20 (9)
Health Professionals	35.6 (16)
Non-Profit Sector	22.2 (10)
Housing Sector	17.8 (8)
Education	4.4 (2)
**Age Group**	**n (%)**
Not given	4.4 (2)
25–29	6.7 (3)
30–34	15.6 (7)
35–39	13.3 (6)
40–44	15.6 (7)
45–49	22.2 (10)
50–54	2.2 (1)
55–59	6.7 (3)
60–64	11.1 (5)
65-69	2.2 (1)
**Gender**	**n (%)**
Not given	2.2 (1)
Male	11.1 (5)
Female	83.7 (39)
**Ethnic Group**	**n (%)**
Not given	6.7 (3)
White (British and/or Any other White British background)	77.8 (35)
Black (African or Caribbean)	6.7 (3)
Asian or Asian British: Indian	6.7 (3)
Mixed Ethnic background	2.2 (1)
**How Long Professionals Have Worked with Families Living in Temporary Accommodation (Time Spans)**	**n (%)**
<1 year	0

1-5 years	26.7 (12)
5-10 years	33.3 (15)
>10 years	40 (18)
**Total**	**45 (100%)**

Roles of participants interviewed

The professionals interviewed in this study represented a diverse range of roles across healthcare, housing, education, and the charity sector, all working to support children under 5 living in TA in England. Healthcare professionals included GPs, health visitors, midwives, nurses, dentists, a paediatrician, and a speech and language therapist, many specialising in homelessness, safeguarding, refugee support, and perinatal care. Housing professionals, including caseworkers, support workers, solicitors, and housing directors, highlighted that they provided advice on housing rights, challenged poor living conditions, and liaised with local authorities. Educators, such as an early years teacher and a nursery director, supported children's development in challenging circumstances. Non-profit professionals including charity directors, coordinators, peer support leads, play workers, policy advisors, and volunteers, highlighted their focus on direct service delivery, emotional and educational support, advocacy, and strategic planning to address the complex needs of families in TA.

Barriers

The results of this study identified a range of barriers affecting professionals' ability to deliver integrated care for children under 5 living in TA. Findings were organised using a multi-level framework that distinguishes between practice-level, organisational, and systemic challenges (Figure 1). This approach highlights how barriers operate at different levels of the service system and interact to shape participants' experiences.

1. Practice level challenges (frontline and individual practice)

1.1 Knowledge and skill limitations

1.1.1 Lack of training and knowledge

The lack of accessible and relevant training was identified by participants as a significant barrier. Professionals highlighted a siloed approach within professional development systems, where they reported limited opportunities to learn how to support families in TA, as well as how to collaborate with other professionals and understand their roles within the wider system:

> *"In terms of ensuring there is adequate care or adequate access for those who are homeless, no training whatsoever." (1132)*

> "*We have a range of a range of expertise within the team, but nobody who's specifically qualified or trained to do work in that specific area…which can be stressful." (3166)*

> "*No, no, not specifically [training], but they [homeless families] do, I would say, cover quite a large proportion of our caseload…One of the things that we find difficult [without training] is negotiating how it works within local authorities to get the support*

For peer review only

and the housing. And what is allowed and what isn't, and yeah it's a bit sticky, it's a bit of a minefield…these things change quite frequently as well, like they do for everything." (3058)

"From what I found when I've looked for training courses for my staff, there is not a lot widely available... especially not in the field of working with children who are facing multiple adversity." (2776)

Instead, teams often relied on informal, experience-based knowledge transfer:

"I think we've probably arrived at that [knowledge about supporting homeless children] by a mix of different things and a bit of learning on the job. But yeah, I'm not aware of anything specific that we've done... Young children especially tend to get a bit forgotten in the whole homelessness issue.. there's a gap and obviously we've got our kind of health visitors and things like that, again, I wouldn't be confident about their level of understanding really when it comes to homelessness because it is a really complicated picture." (4048)

"I wouldn't say I have any specific training at all, it's more just experience and a general interest in looking after people who are I guess more socio economically deprived." (1388)

Moreover, this reported absence of training led to professionals stating they felt unprepared, possibly creating bottlenecks in service delivery particularly during the pandemic. As one participant reflected:

"Our team, we did feel a bit out of our depth at one point." (7114)

1.1.2 Individual constraints to upskill

Another significant reported barrier was the lack of time and capacity within roles to reflect or upskill which prevented professionals from moving beyond reactive, day-to-day duties to adopt a more strategic approach:

"I think it [training] would be useful, it would be useful. I suppose in some sort of format, it's difficult to imagine how that would look for us as GPs, because I just don't know how that would look in terms of time, I suppose." (1388)

"And then we're at the stage now we need a little bit more calm and capacity, a bit more head space. Twenty percent of our staff are off sick at the moment, so you try and put a training program together and it doesn't make sense. They're just on survival mode. So wait for the pandemic to die down and then the recovery period, probably in the spring summer, it's a little quieter, we will then put together, put our heads together. And look at ways in which we can address this. Either through some professional educational input, which goes to everybody, and we have to think again how to do it. But at the moment there's just no space, head space to do it." (4828)

1.2 Interprofessional practice challenges

1.2.1 Misaligned goals between teams

For peer review only - http://bmjopen.bmj.com/site/about/guidelines.xhtml

A fundamental challenge emerged around the differing priorities between teams, which reportedly hindered collaboration and reduced the effectiveness of integrated support. Professionals reported that while health professionals considered housing a critical determinant of health, housing officers focused solely on their legal duties:

> *"Because for a housing officer, it's not really on their agenda, whether they've been for a smear test or whether the baby's had immunisations or whether or not that family is practicing safe sleep, all of these prevention things. It's not really their... it's not their priority. Their priority is to look at the legal duty that has been offered to that family... Housing Officers do not see that health is part of what will solve their problems." (1151)*

This divergence in priorities was compounded by systemic fragmentation between teams:

> *"We all got different funding. We're all different agencies and nobody talks to each other. And that's the key problem. And it's like that with the NHS actually isn't it I mean that the left hand never talks to the right everything needs to come under one umbrella really when it comes to children because it's a whole process. You can't just have one thing being done. And the other bit nuts it's like having a jigsaw and not being able to finish it." (6555)*

Furthermore, professionals described difficulties in securing buy-in from leadership across teams, reinforcing silos in service provision:

> *"Sometimes you're asked during professional development or during your one to one's, what kind of professional development do you need, what will help you better to do your job properly. How can you help our residents better? And I would make suggestions and it's almost like well, we've tried that, we're not getting anywhere because the other team managers are not cooperating. So, it's almost like there's a barrier because we have tried…but you can't get the buy in from the other departments because there's a, it's just as bureaucracy and barriers… If people don't buy into a certain practice, and it comes from leadership really." (2226)*

1.2.2 Poor communication between teams

Limited communication was a recurring issue, reported as contributing to inefficiencies and delays in service provision. Many professionals described that they found themselves acting as intermediaries due to fragmented communication channels:

> *"So then we become sort of mediators between what customer services cannot do and between back-office housing where they don't have that direct contact. Because even the phone lines and the emails, I think due to demand, in my opinion, it's almost like there's a barrier there. They can't access housing advisors, officers, housing officers directly. Unless sometimes by luck, the phone, somebody picks up or somebody decides they're going to email them. Because they might send them one email and six months later they hear back. But they are all irregular or poor communication." (2226)*

> *"the local authority decided to move these children from some of the homeless units to another homeless accommodation, either within the borough or outside the borough. So again, it took a long long time trying to trace these children because they disappear over, we don't know where they moved to." (1988)*

For peer review only

Professionals also highlighted that escalation of their concerns via standard, recommended routes, such as commissioner intervention, were ineffective:

> *"We've even had emails from commissioners saying oh if they do that, if they move somebody without letting you know, if you request for delayed dispersal and they disperse the person [then] this is the person to email. And we continually do that but nothing happens, it still happens, it still happens yeah." (4029)*

2. Organisational challenges (service and workplace context)

2.1. Workforce and capacity constraints

2.1.1 Reduced staffing levels and high staff turnover

Staffing shortages were highlighted as a significant barrier, with professionals describing burnout and high turnover rates as contributing to inefficiencies.

> *"There's a lot of mandatory training you have to do to work for our trust. So the bank staff, we did hire, our recruitment time takes like, three, four months. It's a really, really long, arduous process. So that's the first part. And the second part where they've now asked, Does anyone want to do Saturdays or evenings? Everyone's burnt out - no." (4999)*

Many teams were described as not able to access any additional funding or extra staff to help with the increased workload during the pandemic:

> *"No. If anything it's less [funding], because you'd have to cover, as times gone on people are shielding or not shielding and all the rest of it. You're covering your colleagues work, staff are just ill, they're burned out, they're stressed. So staffing has been a huge, huge issue, much worse. And lots of people have been deployed as well." (5186)*

2.1.2 Workload and capacity issues

High caseloads and limited capacity were key reported concerns, particularly in urgent care settings where referral processes created bottlenecks:

> *"These urgent care centres will receive a walk in or a phone call from a patient asking to be seen, a child, and before even assessing the child they just send them straight to the community referral service. Which then creates a huge backlog for these community dental services. And it's just, it's very, very unreasonable when these children could actually be treated at the urgent care centres." (1132)*

Participants described the backlog in services, which was exacerbated by the pandemic, as further restricting access to routine and preventative care:

> *"Very difficult at the moment, because we have such a huge backlog because of the pandemic, not being able to see patients routinely for so long. We're still getting through our backlog, there are still patients who I've seen this week who haven't been seen for two years and they are registered in our books. So our registration is closed at the moment, so we're not taking on any new patients. So we can see them on an*

For peer review only

emergency basis, but not to take on to give them that preventative care, to give them that routine care. It's just to get them out of pain only." (1132)

2.2 Operational barriers

2.2.1 Data sharing and access issues

Many professionals reported that data protection regulations, such as the UK General Data Protection Regulation (GDPR), were often used as an excuse to restrict access to essential information about children in TA:

*"Internally, we just say GDPR is a b******* excuse because we're talking about safeguarding of vulnerable kids…So, if this was a domestic violence issue and a kid had been beaten up, everyone would know but we put these really vulnerable families in incredibly vulnerable situations in B and Bs with other kind of difficult single adults, or we put them out of borough, or we put them in disgusting accommodation, and suddenly, no one's really bothered… all we want to do is go, tell us who they are and we'll help them, and they just say, Oh no, it's GDPR… if we're talking about safeguarding children, then you need to share the data with us." (3597)*

Participants felt that the consequence of this limitation in access to essential information meant staff often had to take on additional tasks outside their usual responsibilities to obtain the details needed to support families:

"So I then have to do the secondary work of emailing other colleagues to say can you check the data, can you check if you've got any information for the landlord. Other than that, other teams might have access to the land registry, that tells us the type of property and who's the owner, whether they're a leaseholder or sub tenant etc. But again, you need a license for that, and only specific teams have that." (2226)

Moreover, the described that existing data collection processes failed to capture key demographic details about children, limiting the ability to tailor support services effectively:

"They don't even acknowledge the age of children, or the ethnicity of the family on their figures. One, because it's not important to them. Because if it was, it would be being measured… So these things we, we often will say we know how many people are in the household, but don't know the ages of the children. Where is that information being, because if you don't know how many children are in that situation, you can't change it. In that sense, that way the five year old needs to be in school, and that three year old need universal 16 hours of childcare provision, you know, to make sure there's some socialisation going on." (1151)

2.2.2 Limited internal coordination mechanisms

A further barrier identified was the lack of notification when families are relocated which disrupts continuity of care. Participants were concerned that without proper alerts, existing data-sharing systems will fail to support effective interagency collaboration, increasing the risk that vulnerable families are overlooked:

"The biggest barrier there is that no one tells any sort of health professional, any sort of social care professional when a family has declared themself homeless. Yeah, they

For peer review only - http://bmjopen.bmj.com/site/about/guidelines.xhtml

will be placed in temporary accommodation and no one's doing any notification." (6631)

The impact of these data-sharing constraints was described as far-reaching, as many teams describe still receiving referrals through chance conversations rather than structured communication channels:

"I did have a family that transferred into temporary accommodation and we didn't know via housing or anything like that. It's only because the family nurse partnership team in that area contacted us and said oh did you know, and we were like okay, we'll take them on then." (3058)

Furthermore, participants reported that just as families are connected to support services, they are often relocated before they can fully benefit from them:

"They come in and they go out and by the time somebody finds them they're about to go out. And they might be in there for weeks on end of having no services. By the time, this is my colleagues, they've got the services up and running they're just about to move out so they're not there to receive all the lovely services." (1663)

Participants were concerned ensuring continuity of care for these families largely depended on the dedication and goodwill of individual professionals rather than a reliable information sharing system:

"It just relies on the goodwill of the individual practitioner, because as I said during this pandemic I could have just not done anything. Or I could have done something that I thought was, in many ways, helpful to my clients. Yeah, so it's not something that I would have been kind of expected to, if I couldn't, I wouldn't have been expected to do, I didn't have to do it." (1988)

3. Systemic and institutional challenges (broader policy and system context)

3.1. Policy and funding constraints

3.1.1 Lack of funding and senior support

Professionals highlighted how funding structures and reliance on senior support at the institutional level posed barriers to their ability to deliver integrated, preventative care for young children in TA. In particular, professionals highlighted the way service payment models in dentistry actively disincentivised early intervention:

"If you have a child under the age of two, you're not really going to be doing any treatment on them. So, the dentist, the dental practice doesn't get paid to have that preventative chat with the patients. So there's no financial incentive of seeing patients under the age of two, so I think that dentists don't see patients under the age of two." (1132)

In examples where effective integrated care was successful, participants noted that such initiatives were often dependent on charitable funding rather than sustained statutory investments:

For peer review only

"We got some money from the mayor's charity. So they gave us quite a considerable amount to help with trauma support work. They were doing, like, safeguarding training with the b&b staff and I kind of set up a little hub of connected professionals that all working together so we kind of were doing that kind of work, and then we've got money from the Morrison's CEO…to help homeless families in a large metropolitan area in the North of England. We provided hot meals in the B and Bs for a few months at the start of COVID. We did care packages we did, and cleaning packages and all that kind of stuff, all that money was paid on that. We commissioned a local restaurant to do hot food for all the families in b&bs. So I think we did 2000 meals over 3-4 months for all the families. And all that money kind of was, was spent on doing that." (3597)

"Our average house was decommissioned by the local authority, but we just felt as a charity it was a project that we just couldn't stop doing because it was always full. And there was a need there and we felt we're making a real difference for the families that did come through. And so we've been able to, you know, our boards very supportive of bridgehouse. So we were able to continue that and use some of our charity reserves and do some fundraising to make sure that that was able to continue." (4048)

Participants also highlighted that they felt that preventative care was hindered by a reliance on strategic leadership buy-in at the institutional level:

"*Unless the officer, the managers and the, the higher, the chief officers, are aware that we need to do some prevention work to stop these families coming back into the homeless system - what we know is if babies and children are in the homeless system we know that they'll be more likely to be adults that will be within the homeless system. So it's really the open mindedness of those strategic leads to see that if we can implement and step in with prevention.*" (1151)

Another point raised was the potential success of homeless health visitors to integrating care; however, participants highlighted how policy specifications and a lack of senior-level support limited the adoption of such roles locally:

"So I think that, and I am actually trying to fight for it, for there to be a homeless self visitor for our area. Particularly because of the Afghan refugees, we've suddenly got like 600 families all moved in. And they were very, our borough they were very against that, they weren't going to have it, but it's gone down really well in various areas including other boroughs. When I suggested it, they said it's not part of the spec for this area, so no." (5186)

3.1.2 Policy constraints and catchment area limitations

Service provision was described as highly inconsistent due to catchment area policies:

"So some children you just lose track of them because they don't ever get back…when it's impossible, for whatever reason, in exceptional circumstances we will retain that

For peer review only

care just to keep the continuity going for the child. Despite the fact that it might be outside a contract of some sort. These are the constraints that really these poor families in a way are up against, institutional constraints. Not because the clinician doesn't want to see them but because the institution rules are such that they've now moved out, they need to go somewhere else… we've been prohibited by an institutional rule as opposed to what's clinically in the best interest of the child." (4828)

The data suggested that differences in eligibility criteria between local authorities and charities created frustration, as some individuals fell outside the support systems in place:

"There's obviously cases where the local authority, particularly, that obviously they have an eligibility criteria, and some of the people that we support will at times fall outside of that where there can be frustration, I think, as a charity, because we kind of want to help anybody who's experiencing homelessness, and the local authority and some other charities in the city have different criteria around who they work with." (4048)

3.2 Service system gaps

3.2.1 Lack of specialised services across the system

Participants were concerned that the absence of dedicated services for families in TA meant that existing services often failed to meet their needs:

"I don't think one could be optimistic unless you redesign the service for those families. You have to understand their needs and I think health visitors are in a fantastic position to do that. And then you have to be able to define a response to those needs that actually is contracted specifically. It's not going to happen within the existing services, they will fail. We will fail these families, I know that." (4828)

Participants described specialist outreach and dedicated teams as highly effective in improving integrated care, yet their reflections also point to the absence of sufficient specialist capacity as an ongoing barrier to embedding these models more widely:

"I'm very heavily involved in improving integrated care systems so that there's [specialist] outreach to general practice…And my experience of that has been brilliant because the parents absolutely love it. And I had a parent in tears, who had spent six years trying to get a specialist appointment…And it's professionally very satisfying because actually they all turn up, everybody knows where their GP is. And in terms of vulnerability and acceptability, its like they're on, you know, this is nearer to my home turf. There are lots of advantages but lots of disadvantages to the profession, in terms of travel time." (4828)

"Health visitors are just leaving in droves and staffing issues are just getting worse and worse and worse. So this [having a specialist team] would be a good way to attract people into the profession and to retain the ones that are there. So I think having a specialist team would be really good." (5186)

For peer review only

DISCUSSION

To our knowledge, this is one of only a few qualitative studies, set in a high-income country, which examines cross-sector professionals' experiences of delivering integrated care to children under 5 living in TA during the COVID-19 pandemic. This study builds on existing research highlighting barriers to accessing care faced by families from professionals' perspectives [12]. Many of the organisational and systemic barriers identified in this study, such as fragmented service provision, workforce instability, poor communication, and limited data sharing, have been well documented in the literature [20-23]. These challenges therefore cannot be understood as direct consequences of the COVID-19 pandemic. Rather, the pandemic acted as a stress test that intensified pre-existing weaknesses, rendering long-standing structural failures more visible, and, in some cases, more acute. While the pandemic motivated innovative and opportunistic service integration to meet demands, it also exposed the fragility and inequity of existing systems, which had long lacked the infrastructure needed for sustainable collaboration. The findings of this study reveal a complex web of systemic challenges, including siloed team structures, inadequate communication, lack of specialised training, resource constraints, and restrictive institutional policies - the majority of which remain barriers today [41]. This study highlights how these barriers operate across individual, team, work environment, and institutional levels.

At the practice (individual) level, a key barrier was the lack of formal training for professionals, with many feeling unprepared and relying on experiential rather than structured evidence-based learning. Similar to previous research [42,43], the absence of training resources increased staff stress and lowered confidence. Limited professional development confined staff to reactive crisis management, reducing capacity for sustainable support and skill-building. This reactive focus aligns with previous research, which found that services working with vulnerable populations are frequently crisis-driven and tend to diminish once immediate risks subside [44]. Embedding tailored training into routine practice is essential to improve service continuity and workforce preparedness [45]. While inadequate training and reliance on experiential learning are long-standing issues in service provision, the pandemic intensified their consequences by accelerating redeployment, increasing caseload complexity, and reducing access to informal learning and supervision, thereby amplifying professional stress and reactive practice.

At the organisational and team level, conflicting priorities and poor communication, particularly between health and housing services, were significant barriers to integrated care, often sidelining the child's needs. These challenges led to inefficiencies, delays, and fragmented services, consistent with previous findings on cross-sector collaboration [44,46]. Professionals often took on extra coordination tasks to bridge communication gaps and prevent families from falling through service gaps, increasing their workload and detracting from direct service delivery - a response similarly noted in a study on healthcare for vulnerable populations [47]. Staffing shortages further disrupted service continuity, hindered relationship-building, and weakened collaboration, echoing existing research highlighting the impacts of workforce instability [48]. Although workforce instability and high turnover were well-recognised challenges prior to the pandemic [20-23], COVID-19 exacerbated these trends through staff illness, burnout, emergency redeployment, and rapid organisational changes, significantly reducing opportunities for relationship-building and coordinated working. The pandemic further strained already understaffed services [49], leaving professionals in a constant state of "firefighting" and contributing to widespread burnout [50,51]. Post-pandemic challenges persist, with housing providers frequently hiring at pace to fill urgent gaps, often prioritising speed over skills and experience [52]. This reactive approach not only affects service quality

but also makes it harder to foster the stable, long-term professional relationships needed for effective, child-centred care.

Within the work environment, workload pressures led to teams operating in silos, with poor collaboration creating inefficiencies and backlogs. This often resulted in delayed referrals and missed opportunities for children in TA to access vital health and support services. While siloed working across housing and social sectors supporting homeless populations is well-documented [18,53,54], this study demonstrates how pandemic-related pressures worsened not only cross-sector fragmentation, but also fragmentation within healthcare teams themselves, highlighting a less frequently examined barrier to integrated care for children in TA.

One of the most significant operational barriers to integrated care was the restriction of data sharing between teams, limited internal coordination mechanisms and thus the inability to identify and track access and outcomes over time and geography. The lack of interoperable IT systems is a well-documented challenge for integrated homeless services, limiting cross-sector collaboration [55]. A 2015 survey by the Health Service Journal found that over 60% of professionals believed that data protection regulations were hindering their efforts to integrate health and social care services [56]. Our findings also reveal that homeless children are often excluded from data-sharing structures designed to safeguard vulnerable groups, highlighting how current systems overlook their needs - an issue made especially visible during the pandemic, when there was a greater reliance on digital systems, and during a time of heightened risk for children [57]. Greater legislative flexibility is needed to enable secure, efficient information sharing [44], especially for children in their formative years, as their frequent relocations increase the risk of them slipping through the gaps in care [58,59]. Enabling local systems to link and share data, professionals can gain a more complete picture of the health and social needs of homeless children [60].

At the systemic level, funding constraints, particularly limited investment in prevention, was noted as a barrier to integrated care, aligning with existing research (61–63). Children under 5 in TA, already at a disadvantage, feel the impact of this most acutely, with the pandemic further widening health inequalities [12]. The lack of investment in prevention not only undermines children's long-term health outcomes but also places greater strain on already overstretched services [63]. System-level differences in catchment areas and eligibility criteria among services posed further challenges to integrated care. These inconsistencies often lead to unnecessary duplication of services, as highlighted by the Institute for Government which found that overlapping responsibilities can result in inefficiencies [30]. More critically, this study found that such misalignments frequently disrupt continuity of care, causing vulnerable families to fall through the gaps in siloed support systems. These findings suggest that without targeted post-pandemic reform to address long-standing systemic barriers, rather than temporary crisis responses, services risk reverting to pre-pandemic patterns of fragmented care, with enduring consequences for children under 5 living in TA.

Strengths

A key strength of this study was the combination of diverse cross-sector perspectives, capturing insights from professionals across health, housing, and social care. The national scope offers broad applicability across England and strengthens the transferability of the findings beyond local contexts. The use of semi-structured interviews and detailed topic guide that was informed by larger team's expertise in the area provided consistency across participants while allowing in-depth exploration of individual experiences, gathering rich nuanced insights into

For peer review only

barriers faced by professionals. The research fellow holds a PhD in sociology and is well trained in the use of qualitative research methods. She was closely supervised by lead researchers on the project with in-depth contextual and methodological expertise relevant to this research. Additionally, the use of co-coding and consensus discussions during analysis enhanced the reliability and rigour of the findings, reducing individual bias and ensuring consistency in theme identification [64]. The research also aligns with current national and local priorities, contributing to the recent all-party parliamentary group call for evidence on temporary accommodation and supporting NHS integrated care boards efforts to address health inequalities and homelessness [65]. Finally, the study was supported by a team with significant expertise in health and social care systems research, strengthening the rigour and interpretation of the findings.

Limitations

A limitation of this study is that recruitment was particularly challenging as the study took place during the COVID-19 pandemic, when there were significant service disruptions, staff redeployment, and heightened professional responsibilities. Therefore, availability of some staff was limited, which extended recruitment timeframe and localities of where professionals were recruited from - relying on opportunistic sampling rather than systemic representation of service provision across England. As a result, the study does not account for local variation in service provision or allow for comparative analysis between regions. However, data saturation was reached, indicating that key themes were consistently identified across interviews despite recruitment challenges. Lastly, the data was collected almost 3 years ago, however, the findings remain valuable for informing current and future practices, particularly as services continue to recover from the pandemic and many of the barriers remain relevant.

Recommendations

Strengthening integrated care for children in TA requires targeted cross-sector training to build understanding of population needs and different service roles, improving collaboration and delivery. In addition, a notification system, as proposed by the Shared Health Foundation [57], could alert services when families enter TA, helping prevent children from falling through gaps. In the short-term, investment in key workers, shown effective in the Supporting Families programme [66], would support continuity of care for families facing relocations. Ultimately, insights from this study highlight the urgent need for substantial capital investment in integrated recovery programmes and the adoption of a universal theory driven model of integrated care that prioritises cross-sector collaboration, early intervention, and preparedness for future crises.

Future research

Future research should focus on a longer term post-COVID study to reflect on pandemic learnings and further examine persistent barriers to cross-sector collaboration in supporting children in TA. Future research should also explore how geographical differences affect the delivery and accessibility of support for children in TA. Local variation in resources, services, and inter-agency coordination may significantly influence outcomes and should be examined through comparative, place-based analysis. Lastly, further research focusing on the experiences and outcomes of families themselves would provide valuable insights into the real-world impacts of systemic barriers and help shape more child-centred policies.

For peer review only

CONCLUSION

This study highlights the complex, multi-level barriers that professionals face when delivering integrated care to children under 5 in TA. From siloed working practices and limited training to staffing shortages and restrictive data-sharing policies, these challenges collectively hinder cross-sector collaboration and reflect long-standing issues in service delivery. The findings highlight the urgent need for systemic reforms, including improved cross-sector communication and training initiatives, flexible data-sharing frameworks, and greater investment in integrated recovery plans to support children post pandemic. Leveraging cross-sector expertise and fostering interdisciplinary collaboration can cultivate a more skilled, coordinated workforce, ensuring equitable access to quality care for children under 5 in TA.

For peer review only

Contributors

PP - Data Curation, Analysis, Writing (Original Draft Preparation). NS - Conceptualisation, Funding Acquisition, Methodology, Supervision, Analysis, Writing (Review & Editing). HA - Analysis, Writing (Original Draft Preparation). SA - Supervision, Data interpretation, Writing (Review & Editing). MA - Data interpretation, Writing (Review & Editing). YP - Data interpretation, Writing (Review & Editing). MH - Conceptualisation, Funding Acquisition, Writing (Review & Editing). MU - Conceptualisation, Funding Acquisition, Writing (Review & Editing). ML - Conceptualisation, Funding Acquisition, Project Administration, Methodology, Supervision, Analysis, Writing (Review & Editing) , Guarantor.

Funding

ESRC, as part of UK Research & Innovation's rapid response to COVID-19 (No.ES/V016253/1).

Acknowledgments

The authors wish to thank all of the professionals who took the time out of their busy schedules to take part in these interviews, especially given the challenging circumstances in which data was collected. We would like to acknowledge the hard work of researchers that supported the development of the project ethics and data collection - Dr Margarita Garfias Royo and Dr Kriss Fearon respectively. We would also like to thank all our partner organisations who supported CHAMPIONS and all the participants who kindly gave their time to support the study. We would also like to thank Dr Diana Margot Rosenthal for her contribution to the conceptualisation of the project, writing of the CHAMPIONS project proposal, and sharing of insights from her PhD research on health and healthcare service access for children under 5 living in temporary accommodation due to experiencing homelessness. Finally, the authors would like to thank UKRI/ESRC for funding this important work.

Competing interests

The authors declare no competing interests.

Data availability statement

The data that support the findings of this study are available from the corresponding author upon reasonable request.

References

1. K. Richard, M. and Shinn , M. (2024) ‘Chapter 28: Preventing and ending family homelessness’, in *Research Handbook on Homelessness*. EE Elgar, pp. 374–387.
2. Hart, A. (2025) Record child homelessness statistics, Shelter England. Available at: https://england.shelter.org.uk/what_we_do/updates_insights_and_impact/record_child_homelessness_and_soaring_rough_sleeping_figures#:~:text=Temporary%20accommodation%20at%20an%20all%2Dtime%20high&text=The%20new%20statistics%20reveal%20that,new%20record%20in%20two%20years (Accessed: 01 March 2025).
3. At least 354,000 people homeless in England today (2024) Shelter England. Available at: https://england.shelter.org.uk/media/press_release/at_least_354000_people_homeless_in_england_today_ (Accessed: 01 January 2025).

4. Svirydzenka, N., Lakhanpaul, M. and Williams, J. (2025) *In their own words: Children's experiences in temporary accommodation*, *Shelter Scotland*. Available at: https://scotland.shelter.org.uk/professional_resources/policy_library/in_their_own_words_childrens_experiences_of_temporary_accommodation
5. Homeless in a pandemic: 253,000 people are trapped in temporary accommodation (2020) Shelter England. Available at: https://england.shelter.org.uk/media/press_release/homeless_in_a_pandemic_253000_people_are_trapped_in_temporary_accommodation_#:~:text=Shelter%20investigation%20exposes%20daily%20struggle,highest%20figure%20for%2014%20years (Accessed: 01 January 2025).
6. Wilson, W. and Barton, C. (2023) Households in temporary accommodation (England) - House of Commons Library, UK Parliament. Available at: https://commonslibrary.parliament.uk/research-briefings/sn02110/ (Accessed: 01 January 2025).
7. Rosenthal, D.M. *et al.* (2020) 'Impacts of covid-19 on vulnerable children in temporary accommodation in the UK', The Lancet Public Health, 5(5). doi:10.1016/s2468-2667(20)30080-3.
8. Coughlin, C.G., Sandel, M. and Stewart, A.M. (2020) 'Homelessness, children, and covid-19: A looming crisis', Pediatrics, 146(2). doi:10.1542/peds.2020-1408.
9. Abrams, E.M. *et al.* (2022) 'The COVID-19 pandemic: Adverse effects on the social determinants of health in children and families', Annals of Allergy, Asthma & Immunology, 128(1), pp. 19–25. doi:https://doi.org/10.1016/j.anai.2021.10.022.
10. Tapper, J., Fazackerley, A. and Thorpe, V. (2025) 'The pandemic reinforced existing inequalities – it was a magnifying glass': How covid changed Britain, The Guardian. Available at: https://www.theguardian.com/world/2025/mar/09/the-pandemic-reinforced-existing-inequalities-it-was-a-magnifying-glass-how-covid-changed-britain (Accessed: 13 March 2025).
11. Development matters (2023) GOV.UK - Department for Education. Available at: https://www.gov.uk/government/publications/development-matters--2/development-matters (Accessed: 01 January 2025).
12. Rosenthal, D.M. *et al.* (2023) 'How does living in temporary accommodation and the COVID-19 pandemic impact under 5s' healthcare access and health outcomes? A qualitative study of key professionals in a socially and ethnically diverse and deprived area of London', International Journal of Environmental Research and Public Health, 20(2), p. 1300. doi:10.3390/ijerph20021300.
13. Filip, R. *et al.* (2022) 'Global challenges to public health care systems during the COVID-19 pandemic: A review of pandemic measures and Problems', Journal of Personalized Medicine, 12(8), p. 1295. doi:10.3390/jpm12081295.
14. How providers are working together across systems in response to COVID-19 (2022) Care Quality Commission. Available at: https://www.cqc.org.uk/publications/major-reports/how-providers-are-working-together-across-systems-response-covid-19 (Accessed: 01 January 2025).

15. Bell, L., Whelan, M. and Lycett, D. (2022) 'Role of an integrated care system during COVID-19 and beyond: A qualitative study with recommendations to inform future development', Integrated Healthcare Journal, 4(1). doi:10.1136/ihj-2021-000112.
16. Monitoring the impact of COVID-19 fortnightly homelessness briefing 6: focusing on emergency hotel accommodation (2020) Groundswell. Available at: https://groundswell.org.uk/wp-content/uploads/2020/07/COVID-19-Fortnightly-Briefing-6-FINAL.pdf (Accessed: 01 January 2025).
17. Doran, K.M. and Tinson, A. (2021) 'Homelessness and Public Health: Lessons Learned from the COVID-19 Pandemic ', European Journal of Homelessness, 15(3), pp. 49–62. Available at: https://www.feantsaresearch.org/public/user/Observatory/2021/EJH_15-3/Final/EJH_15-3_A3.pdf.
18. Daly, M., Ryu, S. and Polat, E. (2023) *Child-related policies in the first year of the COVID-19 pandemic in 40 countries*, *UNICEF Innocenti Global Office of Research and Foresight*. Available at: https://www.unicef.org/innocenti/documents/child-related-policies-first-year-covid-19-pandemic-40-countries (Accessed: 01 March 2025).
19. Mann, E. *et al.* (2024) 'A qualitative exploration of the impact of covid-19 on families with a child under 5 years-old in the borough of Newham, East London', *Discover Social Science and Health*, 4(1). doi:10.1007/s44155-024-00082-4.
20. Omerov, P. et al. (2019) 'Homeless persons' experiences of health- and social care: A systematic integrative review', Health & Social Care in the Community, 28(1), pp. 1–11. doi:10.1111/hsc.12857.
21. Siersbaek, R. et al. (2020) Contexts and mechanisms that promote access to healthcare for populations experiencing homelessness: A realist review [Preprint]. doi:10.21203/rs.3.rs-79236/v1.
22. Carmichael, C. et al. (2023) 'Barriers and facilitators to health care access for people experiencing homelessness in four European countries: An exploratory qualitative study', International Journal for Equity in Health, 22(1). doi:10.1186/s12939-023-02011-4.
23. Clark, M. et al. (2021) 'Homelessness and integrated care: An application of integrated care knowledge to understanding services for wicked issues', Journal of Integrated Care, 30(1), pp. 3–19. doi:10.1108/jica-03-2021-0012.
24. McGuinness, K. (2023) Worse than covid - how the cost of living crisis is undermining our hardest hit neighbourhoods, Northumbria Police & Crime Commissioner. Available at: https://northumbria-pcc.gov.uk/v3/wp-content/uploads/2023/05/Worse-than-covid-how-the-cost-of-living-crisis-is-undermining-our-hardest-hit-neighbourhoods-FINAL.pdf (Accessed: 01 January 2025).
25. Arrieta, T. (2022) 'Austerity in the United Kingdom and its legacy: Lessons from the covid-19 pandemic', *The Economic and Labour Relations Review*, 33(2), pp. 238–255. doi:10.1177/10353046221083051.
26. Butler, P. (2024) *The devastating impact covid and austerity had on children in England*, *The Guardian*. Available at: https://www.theguardian.com/society/2024/feb/19/the-devestating-impact-of-covid-and-austerity-on-children-in-england (Accessed: 01 January 2025).

27. Cross, S. *et al.* (2021) ‘Housing, homelessness and children’s Social Care: Towards an urgent research agenda’, *The British Journal of Social Work*, 52(4), pp. 1988–2007. doi:10.1093/bjsw/bcab130.
28. Tu, Y. *et al.* (2024) ‘Interventions to promote the health and well-being of children under 5s experiencing homelessness in high-income countries: A scoping review’, *BMJ Open*, 14(1). doi:10.1136/bmjopen-2023-076492.
29. CHAMPIONS Project (2023). CHAMPIONS Policy Brief, University College London: London. Available at: https://drive.google.com/file/d/1LEYxCNfqnUbgoxnts5Pmvbu50OgvIckn/view
30. Wilson, S. *et al.* (2015) *Joining up public services around local, citizen needs: Perennial challenges and insights on how to tackle them*, *Institute for Government* . Available at: https://www.instituteforgovernment.org.uk/sites/default/files/publications/4564%20IFG%20-%20Joining%20up%20around%20local%20v11c.pdf.
31. Dyakova, M. *et al.* (2021) ‘Placing health equity at the heart of the COVID-19 sustainable response and recovery: Building prosperous lives for all in Wales’, *The Welsh Health Equity Status Report initiative (WHESRi)*. Available at:https://phwwhocc.co.uk/wp-content/uploads/2021/07/WHESRi-Covid-Report-Eng.pdf.
32. Smith, J.A. and Judd, J. (2020) ‘Covid-19: Vulnerability and the power of privilege in a pandemic’, *Health Promotion Journal of Australia*, 31(2), pp. 158–160. doi:10.1002/hpja.333.
33. *Champions Project*. Available at: https://www.championsproject.co.uk/ (Accessed: 01 March 2025).
34. Palinkas, L.A. *et al.* (2013) ‘Purposeful sampling for qualitative data collection and analysis in Mixed Method Implementation Research’, *Administration and Policy in Mental Health and Mental Health Services Research*, 42(5), pp. 533–544. doi:10.1007/s10488-013-0528-y.
35. Parker, C, Scott, S, and Geddes, A (2019) *Snowball Sampling.* SAGE Research Methods Foundations. Available at: https://methods.sagepub.com/foundations/snowball-sampling
36. Taylor, S.J., Bogdan, R. and DeVault, M.L. (2016) *Introduction to qualitative research methods: A guidebook and resource*. Hoboken, N.J: John Wiley & Sons, Inc.
37. Vasileiou, K. *et al.* (2018) ‘Characterising and justifying sample size sufficiency in interview-based studies: Systematic Analysis of qualitative health research over a 15-year period’, *BMC Medical Research Methodology*, 18(1). doi:10.1186/s12874-018-0594-7.
38. Zoom Video Communications Inc. Security Guide-Zoom Video Communications Inc. Available at: https://d24cgw3uvb9a9h.cloudfront.net/static/81625/doc/Zoom-Security-White-Paper.pdf (Accessed: 01 Sept 2021).
39. Braun, V., & Clarke, V. (2006). Using thematic analysis in psychology. Qualitative Research in Psychology, 3(2), 77-101.
40. QSR International Best Qualitative Data Analysis Software for Researchers. NVivo. QSR International. (accessed on 12 December 2023). Available at:

https://www.qsrinternational.com/nvivo-qualitative-data-analysis-software/home?creative=605555104699&keyword=nvivo&matchtype=e&network=g&device=c&gclid=Cj0KCQiAyMKbBhD1ARIsANs7rEEmS573T9mQwlPzfwU-JWgQACMl4I8_lDGdjuOTUU3vyBYIgXXqO5saAvuKEALw_wcB.

41. Hurst , G., Teixeira, L. and Davies, N. (2025) A smarter approach to homelessness, Institute for Government. Available at: https://www.instituteforgovernment.org.uk/sites/default/files/2025-05/A-smarter-approach-to-homelessness.pdf.
42. McNeil, R. et al. (2013) 'Learning to account for the social determinants of Health Affecting Homeless Persons', Medical Education, 47(5), pp. 485–494. doi:10.1111/medu.12132.
43. Cené, C.W. et al. (2010) 'Community-based teaching about health disparities: Combining education, scholarship, and Community Service', Journal of General Internal Medicine, 25(S2), pp. 130–135. doi:10.1007/s11606-009-1214-3. (Accessed: 1st Jan 2025).
44. Klassman, K. et al. (2024) 'A rapid evidence assessment of barriers and strategies in service engagement when working with young people with complex needs', Children and Youth Services Review, 156, p. 107292. doi:10.1016/j.childyouth.2023.107292. (Accessed: 1st Jan 2025).
45. Davis, J. and Lovegrove, M. (no date) *Inclusion Health: Education and training for Health Professionals: Report*, *Allied Health Solutions Limited*. Available at: https://assets.publishing.service.gov.uk/media/5a80ef0040f0b62305b8df8c/NIHB_-_Inclusion_Health_education__Report.pdf (Accessed: 01 January 2025).
46. Black, E.B. et al. (2018) 'Homeless youth: Barriers and facilitators for service referrals', Evaluation and Program Planning, 68, pp. 7–12. doi:10.1016/j.evalprogplan.2018.02.009. (Accessed: 01 January 2025).
47. Pierce, P. et al. (2024) 'Effects of UK hostile environment policies on maternity care for refugees, asylum seekers, and undocumented migrants in Camden: Examining the experiences of healthcare professionals and community organisations', Journal of Migration and Health, 11, p. 100291. doi:10.1016/j.jmh.2024.100291.
48. Phillips, A., Bush, L. and Walcott, S. (2023) Wall to wall support joining up public services and housing for vulnerable children, young people and families. Report. Available at: https://www.grosvenor.com/getattachment/f3ed168a-0fbd-4e83-ab80-f55d6bd67e95/Wall-to-Wall-Support-final-version-EMBARGOED-UNTIL-07-12-23.pdf (Accessed: 01 January 2025).
49. Ofsted (2022) Ofsted: Concerns over damaging impact of staff shortages on children's Social Care, GOV.UK. Available at: https://www.gov.uk/government/news/ofsted-concerns-over-damaging-impact-of-staff-shortages-on-childrens-social-care#:~:text=The%20report%20finds%20that%20the,staff%20are%20able%20to%20support (Accessed: 01 January 2025).
50. Frontline Network (2023) Experiences of frontline homelessness work 2022. rep. Available at: https://www.frontlinenetwork.org.uk/media/2794/frontlinenetworksurvey-workforcefindings-1603.pdf (Accessed: 01 January 2025).

51. Flores, E.V. (2023) Organizational strategies to improve retention among the homeless sector workforce: A Systematic Literature Review. California State University, Long Beach ProQuest Dissertations & Theses. Available at: https://www.proquest.com/openview/525a631b9ed30aeb9372088a417d83df/1?cbl=18750&diss=y&pq-origsite=gscholar
52. The National Housing Federation (2024) The Better Social Housing Review. Report. Available at: https://s46834.pcdn.co/wp-content/uploads/BSHR_Report_FINAL_embargoed_until_Tues13thDec.pdf
53. Cornes, M. , Joly, L. , Manthorpe, J. and O'Halloran, S. (2011), *Rethinking Multiple Exclusion Homelessness: Implications for Workforce Development and Interprofessional Practice (Summary of Findings)*, The King's College London, London. Available at: https://insight.cumbria.ac.uk/id/eprint/4171/1/OHalloran_RethinkingMultiple.pdf
54. Morgan Diamond, M. (2020) 'Pathway to Opportunity: Apprenticeships and Breaking the Cycles of Poverty for Young People Experiencing Homelessness', Young Invincibles. Available at: https://younginvincibles.org/wp-content/uploads/2020/08/IL_Apprenticeship_Pathway_Final_2-min.pdf
55. Ní Cheallaigh, C. (2021) 'Barriers and facilitators of an integrated, Interdisciplinary Inclusion Health Service in Dublin, Ireland.', International Journal of Integrated Care, 21(S1), p. 112. doi:10.5334/ijic.icic20463.
56. Dalton, R. (2017) Integration: Local government and health leaders prefer a whole system approach, Health Service Journal. Available at: https://www.hsj.co.uk/supplement-archive/integration-local-government-and-health-leaders-prefer-a-whole-system-approach/5086572.article#.VhKmJvlVhBc (Accessed: 01 January 2025).
57. Families experiencing homelessness: Notification system (2020) Shared Health Foundation. Available at: https://sharedhealthfoundation.org.uk/uncategorized/families-experiencing-homelessness-notification-system/ (Accessed: 01 January 2025).
58. Russell, M. and Lister, R. (2020) Moving Always Moving. rep. The Children's Society. Available at: https://www.childrenssociety.org.uk/sites/default/files/2020-10/Moving-Always-Moving-Report.pdf (Accessed: 01 January 2025).
59. Mettle-Olympio, B. (2024) Temporary accommodation in London: Causes, consequences, and solutions, Capital Letters. Available at: https://capitalletters.org.uk/temporary-accommodation-in-london-causes-consequences-and-solutions/#:~:text=Children%20living%20in%20temporary%20accommodation%20are%20more%20likely%20to%20experience,stress%20of%20their%20living%20situation (Accessed: 01 January 2025).
60. Caswell, R. (2021) Homelessness and the response to COVID-19: learning from lockdown Data and data sharing, Strategy Unit NHS. Available at: https://www.strategyunitwm.nhs.uk/sites/default/files/2021-02/Homelessness%20data%20and%20data%20sharing%20Strategy%20Unit%20Final%20Report.pdf (Accessed: 01 January 2025).

61. Hughes, G., Shaw, S.E., Greenhalgh, T. (2020) ‘Rethinking integrated care: A systematic hermeneutic review of the literature on Integrated Care Strategies and concepts’, The Milbank Quarterly, 98(2), pp. 446–492. doi:10.1111/1468-0009.12459.
62. Wood, C., Salter, J. and Cheetham, P. (2012) Under one roof. rep. Demos. Available at: https://demos.co.uk/wp-content/uploads/2012/05/Under_one_roof_-_web.pdf (Accessed: 01 January 2025).
63. Curtis, P., Glover, B. and O'Brien, A. (2023) The preventative state rebuilding ur local, social and civic foundations. rep. Demos. Available at: https://demos.co.uk/research/the-preventative-state-rebuilding-our-local-social-and-civic-foundations/ (Accessed: 01 January 2025).
64. Cole, R. (2023). Inter-Rater Reliability Methods in Qualitative Case Study Research. Sociological Methods & Research, 53(4), 1944-1975. https://doi.org/10.1177/00491241231156971
65. Procter, A., Pratt, S. and Wise-Martin, I. (2023) Call for evidence findings: Summary, analysis of themes and call to action, *APPG for Households in Temporary Accommodation*. Available at: https://householdsintemporaryaccommodation.co.uk/reports/call-for-evidence-findings-summary-analysis-of-themes-and-call-to-action/ (Accessed: 01 March 2025).
66. MacAlister, J. (2021) The Case for Change. rep. The independent review of children’s social care. Available at: https://fdac.org.uk/wp-content/uploads/2021/06/case-for-change.pdf (Accessed: 01 January 2025).

FIGURE TITLE

Figure 1. Summary of the themes and sub-themes representing barriers to integrated care

For peer review only

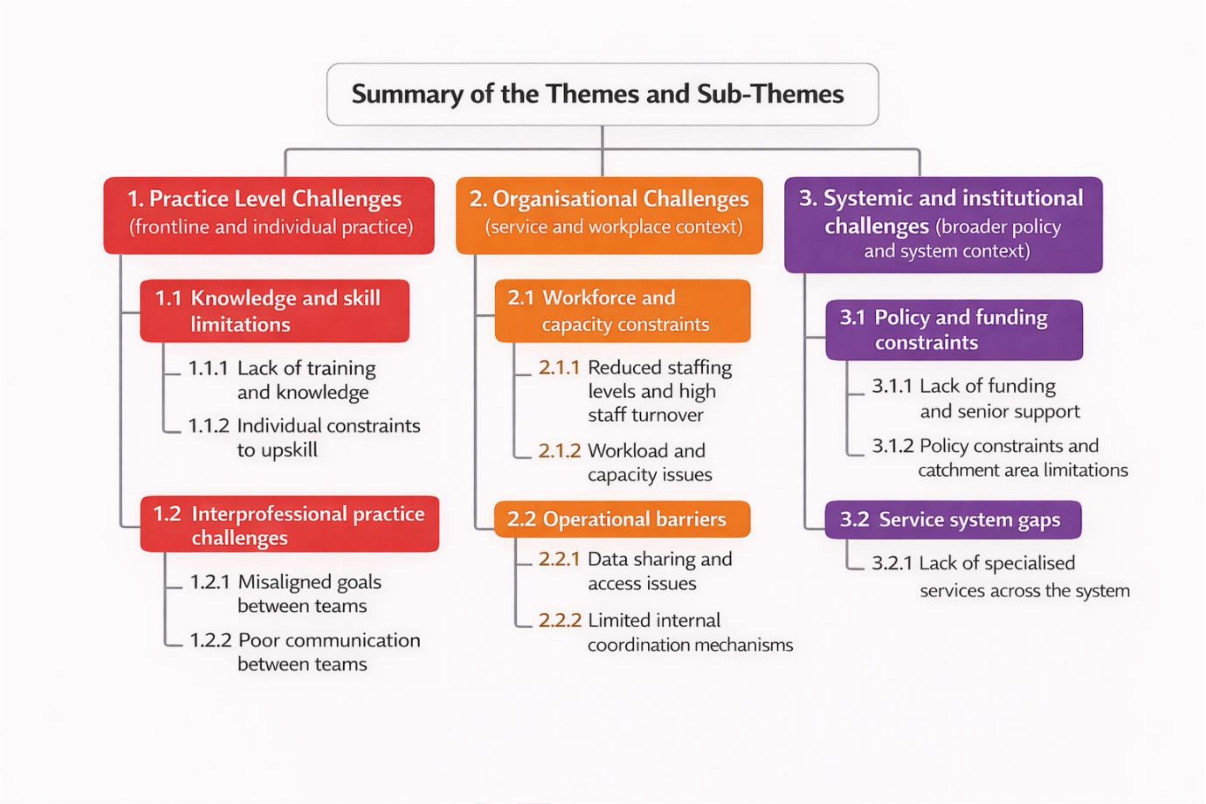
Summary of the Themes and Sub-Themes
1. Practice Level Challenges (frontline and individual practice)
1.1 Knowledge and skill limitations
1.1.1 Lack of training and knowledge
1.1.2 Individual constraints to upskill
1.2 Interprofessional practice challenges
1.2.1 Misaligned goals between teams
1.2.2 Poor communication between teams
2. Organisational Challenges (service and workplace context)
2.1 Workforce and capacity constraints
2.1.1 Reduced staffing levels and high staff turnover
2.1.2 Workload and capacity issues
2.2 Operational barriers
2.2.1 Data sharing and access issues
2.2.2 Limited internal coordination mechanisms
3. Systemic and institutional challenges (broader policy and system context)
3.1 Policy and funding constraints
3.1.1 Lack of funding and senior support
3.1.2 Policy constraints and catchment area limitations
3.2 Service system gaps
3.2.1 Lack of specialised services across the system

CHAMPIONS Interview schedule, Professionals v0-6

Opening

- Personal introduction to researcher
- Interview topics to be covered and focus of interview – ie what is different about living in COVID times compared to living in TA before this
- Interview length, format and breaks
- Confidentiality and consent
- Any questions

Scene-setting

Please tell me a little bit about your role:

- What services do you provide to families experiencing homelessness? Is this service specifically for families or also for other groups?
- Does your area have any services specifically for children?
- How do you work with them under normal circumstances?
- How much experience do you have in working with families living in TA? How do you find out about the specific set of problems these children might face?
- What training do staff have received that is relevant to the group they work with. [If they identify anything] Is this provided in house or externally? Did you source it yourself or is it offered by your employer?
- Are you or other members of your team trained or skilled specifically to work with a) children, b) children in TA?
- Are you familiar with your families' accommodation? Do you have any comments about the quality and safety of the environment?

Focus on specific topics

[Follow up questions will be asked to further explore the key topics of interest where they are not covered already by the discussion of photos]

1. Contact and links with other services

Is families' or the child's homelessness status relevant to the service you provide? If so, what do you do differently with them that you don't do with other families, children or client groups?

Can you tell me a bit about how children in your area come to light to the different services and which services? Which organisation or person is responsible for looking after families / children living in TA? Who keeps track of families? What happens if families move?

Are children in TA referred to you: eg is it flagged on an IT system; are you informed, or do you need to ask?

What are the barriers to finding out about their homelessness status and providing appropriate services? (knowledge, skills, access to data, communication internally or with other agencies)

Are there other barriers to providing a service to this group of children and their families? Do you know of any barriers that families experience in accessing your service?

Is your service part of a pathway for families/children to access your service? Who refers them? Do you refer them on to other agencies, and which ones? Do you work in tandem, and how effective is

For peer review only - http://bmjopen.bmj.com/site/about/guidelines.xhtml

that? Who follows up to ensure that families continue to access services?

How do these various services link together?

2. COVID Knowledge:

How does your organisation find out about COVID restrictions, and keep up to date with changes? How useful were the sources of information and how well did they apply to your service and service users? Who makes decisions about how to define and when to use safe working practices? How are staff informed about this?

Are you or your team responsible for cascading information about COVID restrictions to families, and how do you go about that? How well has that worked? What worked/what didn't?

3. How has COVID changed the way you work?

We began by asking how you normally go about providing your service.

To what extent do you know, based on your experience, how different are the challenges under covid? How has this changed in terms of:

- Access to families or children to provide your service and the length of time it takes
- Access and referrals to other services such as health visitors, medical care, development checks, dental care, ongoing care for physical/mental health issues
- The way you assess their needs.
- Has the assessment method changed?
    - How have families/children's needs changed since covid? How has your service responded to that?
- Are any of these changes likely to become 'business as usual' for your service delivery?
- Has there been a difference in service delivery between full lockdown and living with COVID restrictions?
- Have you been able to access any additional funding or resources (e.g. staff) to support the service change needed to deal with the pandemic? If so how did that come about, was it a request or it came from (local) government? Do you think it will be sustained beyond 2021?

Do you have any recommendations for what could be done to address these issues?

4. What impact is COVID having on families living in TA? → with examples if possible

From your knowledge of the families, what do you think their biggest concerns have been?

Have these issues affected their ability to engage with [your service]?

What do you think has been the greatest impact on children under 5 living in TA through the COVID pandemic and how has it affected their [health/wellbeing and development/education]? What have your main worries / concerns/observations been?

- Child development (health and social aspects)
- Housing quality/issues and access to (safe) outdoor space/play areas
- Nutrition: cooking and eating
- Education
- Wellbeing/Resilience in general

For peer review only - http://bmjopen.bmj.com/site/about/guidelines.xhtml

- Factors that complicate the situation or make it more difficult, such as disability

Are you aware of the local TA boards within their own LA or borough which discuss how to set up and manage services for homeless families? Is there one locally? Is your service involved with it?

Health visitors:

Thinking about the targets set in the Healthy Child Programme, do you think the pandemic has affected the child's:

- mental health
- physical development
- emotional wellbeing
- diet and access to healthy food

and how? What are the priority areas for recovery?

What support do families need to ensure this happens, and which organisations do you need to work with to provide it?

Health professionals:

Charities:

How much of your service provision relies on fundraising, and has COVID affected this?

Has there been any discussion of the financial and wider sustainability of services? Have any decisions been made about changing the way the service is provided?

Housing teams:

How have you found out about the specific needs of people who use your service?

Have your families needed any additional support from your service, such as

- Signposting or support to access welfare services
- Food banks
- Mental health support

What happens to the child or family after they move on from your service?

What happens if the TA accommodation allocated to a family with children is unsuitable for their needs, for example is overcrowded, has dampness/mould, is in need of repairs, has access issues for prams/buggies, is shared with other unsuitable residents: how would you become aware of these problems (e.g. families complain, or regular property inspections) and what are the options available to you for re-housing them and/or making repairs if appropriate?

Education:

Do you know which pupils are living in TA? Have you identified any specific needs for this group?

Are there any services or approaches that you take to support them?

(access to outdoors and spaces to play)

How has COVID 19 affected the education and school-readiness of this specific group? Does the

For peer review only - http://bmjopen.bmj.com/site/about/guidelines.xhtml

school provide additional support (one to one sessions, food parcels, uniform bank, access to IT, printed work pack etc)

Have they been able to access online learning – and what are the barriers to that?

What support do children need during pandemic recovery and afterwards?

Potential solutions

We have discussed your service runs, and how it supports families through the system, and how that has changed during COVID. I want to ask your views on how we can provide a better service for the future, and we set up a system that supports these children.

1. Earlier in our conversation you identified some major issues [such as...] for professionals in your field who are supporting families. What action do you think would address these concerns?
2. We have seen many new innovations and strategies during COVID such as the vaccination bus, the vaccination camps etc. How can we reach families who sometimes find it hard to access services ie how do we bring them services they need closer to them?
3. We are hearing stories about children being 'left behind' following the COVID restrictions, for example low weight, delayed language development and immunisations, and early signs of mental health problems, and there's a view we will need to help children catch up or address problems arising from COVID restrictions. What do you think we should be doing for these children to help them catch up and overcome some of the problems they have gone through?
4. At a national level:
    a. what do you think we should be advocating for to support children - what should our 'ask' be from health, housing, education, local government and central government?
    b. What are the top three actions, or services/facilities that need to be put in place, that you would like to see?
    c. What are your views on setting up an integrated care package around each child?
    d. What are your views on setting up a national notification system?
    e. Who are the key stakeholders in this process?

Closing

Is there anything else you would like to add that we haven't discussed so far?

If you'd like to add any further thoughts or comments, then please do so by contacting me at the email address or postal address on the information sheet.

At the end of the project we will write a summary of the results and send you a copy if you wish.

Thank you for taking part in this research.

For peer review only - http://bmjopen.bmj.com/site/about/guidelines.xhtml

COREQ (COnsolidated criteria for REporting Qualitative research) Checklist

A checklist of items that should be included in reports of qualitative research. You must report the page number in your manuscript where you consider each of the items listed in this checklist. If you have not included this information, either revise your manuscript accordingly before submitting or note N/A.

Topic	Item No.	Guide Questions/Description
Interviewer/facilitator	1	Which author/s conducted the interview or focus group? **Methods, Materials section. Acknowledgments section.**
Credentials	2	What were the researcher's credentials? **Methods, Materials section.**
Occupation	3	What was their occupation at the time of the study? **Methods, Materials section.**
Gender	4	Was the researcher male or female? **N/A**
Experience and training	5	What experience or training did the researcher have? **Methods, Materials section.**
Relationship established	6	Was a relationship established prior to study commencement? **Methods, Materials section.**
Participant knowledge of the interviewer	7	What did the participants know about the researcher? e.g. personal goals, reasons for doing the research. **Methods, Materials section.**
Interviewer characteristics	8	What characteristics were reported about the interviewer/facilitator? e.g. Bias, assumptions, reasons and interests in the research topic. **Methods, Materials section.**
Methodological orientation and Theory	9	What methodological orientation was stated to underpin the study? e.g.grounded theory, discourse analysis, ethnography, phenomenology, content analysis. **Analysis section.**
Sampling	10	How were participants selected? e.g. purposive, convenience, consecutive, snowball **Methods, Participants section.**
Method of approach	11	How were participants approached? e.g. face-to-face, telephone, mail, Email. **Methods, Participants section.**
Sample size	12	How many participants were in the study? **Results section.**
Non-participation	13	How many people refused to participate or dropped out? Reasons? **N/A**
Setting of data collection	14	Where was the data collected? e.g. home, clinic, workplace **Methods, Materials section.**
Presence of non-participants	15	Was anyone else present besides the participants and researchers? **Methods, Materials section.**
Description of sample	16	What are the important characteristics of the sample? e.g. demographic data, date demographic data. **Results section.**
Interview guide	17	Were questions, prompts, guides provided by the authors? Was it pilot-tested? **Methods, Materials section.**
Repeat interviews	18	Were repeat interviews carried out? If yes, how many? **Methods, Materials section.**
Audio/visual recording	19	Did the research use audio or visual recording to collect the data? **Methods, Materials section.**
Field notes	20	Were field notes made during and/or after the interview or focus group? **Methods, Materials section.**
Duration	21	What was the duration of the interviews or focus group? **Methods, Materials section.**
Data saturation	22	Was data saturation discussed? **Methods, Participants section.**
Transcripts returned	23	Were transcripts returned to participants for comment and/or correction? **N/A**

For peer review only

Topic	Item No.	Guide Questions/Description
Number of data coders	24	How many data coders coded the data? **Analysis section.**
Description of the coding tree	25	Did authors provide a description of the coding tree? **Analysis and Results section.**
Derivation of themes	26	Were themes identified in advance or derived from the data? **Analysis section.**
Software	27	What software, if applicable, was used to manage the data? **Analysis section.**
Participant checking	28	Did participants provide feedback on the findings? **N/A**
Quotations presented	29	Were participant quotations presented to illustrate the themes/findings? Was each quotation identified? e.g. participant number **Results section.**
Data and findings consistent	30	Was there consistency between the data presented and the findings? **Results and Discussion section.**
Clarity of major themes	31	Were major themes clearly presented in the findings? **Results section.**
Clarity of minor themes	32	Is there a description of diverse cases or discussion of minor themes? **Results section.**

Developed from: Tong A, Sainsbury P, Craig J. Consolidated criteria for reporting qualitative research (COREQ): a 32-item checklist for interviews and focus groups. *International Journal for Quality in Health Care*. 2007. Volume 19, Number 6: pp. 349 – 357

Once you have completed this checklist, please save a copy and upload it as part of your submission. DO NOT include this checklist as part of the main manuscript document. It must be uploaded as a separate file.

For peer review only
